# Integrative modeling reveals key chromatin and sequence signatures predicting super-enhancers

**DOI:** 10.1038/s41598-019-38979-9

**Published:** 2019-02-27

**Authors:** Aziz Khan, Xuegong Zhang

**Affiliations:** 1 MOE Key Lab of Bioinformatics/Bioinformatics Division, BNRIST (Beijing National Research Center for Information Science and Technology), Department of Automation, Tsinghua University, Beijing, 100084 China; 2 Centre for Molecular Medicine Norway (NCMM), Nordic EMBL Partnership, University of Oslo, 0349 Oslo, Norway; 3 School of Life Sciences, Tsinghua University, Beijing, 100084 China

## Abstract

Super-enhancers (SEs) are clusters of transcriptional enhancers which control the expression of cell identity and disease-associated genes. Current studies demonstrated the role of multiple factors in SE formation; however, a systematic analysis to assess the relative predictive importance of chromatin and sequence features of SEs and their constituents is lacking. In addition, a predictive model that integrates various types of data to predict SEs has not been established. Here, we integrated diverse types of genomic and epigenomic datasets to identify key signatures of SEs and investigated their predictive importance. Through integrative modeling, we found Cdk8, Cdk9, and Smad3 as new features of SEs, which can define known and new SEs in mouse embryonic stem cells and pro-B cells. We compared six state-of-the-art machine learning models to predict SEs and showed that non-parametric ensemble models performed better as compared to parametric. We validated these models using cross-validation and also independent datasets in four human cell-types. Taken together, our systematic analysis and ranking of features can be used as a platform to define and understand the biology of SEs in other cell-types.

## Introduction

Enhancers are *cis*-regulatory regions in the DNA that not only augment the transcription of associated genes but also play a key role in cell-type specific gene expression^1,2^. A myriad of transcription factors (TFs) bind to enhancers and regulate gene expression by recruiting coactivators and RNA polymerase II (RNA Pol II) to target genes^3–7^. A typical mammalian cell is estimated to have thousands of active enhancers, a number which rises to roughly one million in the human genome^1,8^. It has been more than three decades since the first enhancer was discovered^9^, but our understanding of the mechanisms by which enhancers regulate gene expression is still limited. However, development of methods such as chromatin immunoprecipitation followed by sequencing (ChIP-seq) and DNase I hypersensitivity followed by sequencing (DNase-seq), have helped to discover and characterize enhancers at a genome-wide scale. Many factors have been associated with enhancer activity, including mono methylation of histone H3 at lysine 4 (H3K4me1), acetylation of histone H3 at lysine 27 (H3K27ac), binding of the coactivator proteins p300 and CBP, and DNase I hypersensitivity sites (DHSs)^8,10,11^. By exploiting these factors and other genomic features many supervised and unsupervised machine learning approaches have been developed to predict enhancers genome-wide^4,12,13^.

The Mediator is a transcriptional coactivator, forms a complex together with Cohesin to create cell-type specific DNA loops, facilitating enhancer-bound TFs to recruit RNA Pol II at the promoters of target genes^14,15^. The genomic regions co-bound by the embryonic stem cell (ESC) pluripotency TFs Oct4, Sox2, and Nanog (OSN) have shown robust enhancer activity^16^. By using ChIP-seq data for Oct4, Sox2, and Nanog in ESC, 10,227 co-bound regions have been identified and classified into super-enhancers (SEs) and typical enhancers (TEs), ranked by Mediator subunit Med1 signal^17^. SEs are clusters of enhancers, which are cell-type specific, associated with key cell identity genes, and linked to many biological processes which define the cell identity^17^. These SEs are densely loaded with the Mediator complex, master TFs, and chromatin regulators^17–20^. Many disease- and trait-associated single nucleotide polymorphisms (SNPs) have been found in these regions^18^. SEs differ from TEs in terms of size, ChIP-seq density of various cofactors, DNA motif content, DNA methylation level, enhancer RNA (eRNA) abundance, ability to activate transcription, and sensitivity to perturbation^17–19,21–23^. Furthermore, studies have found SEs involved in multiple cancers and demonstrated their importance in cellular-identity and disease and emphasizing their use as potential biomarkers^18,19,24–26^. Other parallel studies have shown similar patterns by using different approaches and termed them ‘stretch enhancers’^27^ and ‘enhancer clusters’^28^.

Since the discovery of SEs, the research community used ChIP-seq data for different factors to differentiate SEs from TEs in different cell-types. ChIP-seq data for Med1 optimally separated SEs and TEs by comparing it with H3K27ac, H3K4me1 and DHSs^17^. BRD4, a member of the BET protein family, was also used to distinguish SEs from TEs as it is highly correlated with MED1^19^. H3K27ac was extensively used to create a catalogue of SEs across 86 different human cell-types and tissues due to the availability of ChIP-seq data for H3K27ac^18^. Other studies used the coactivator protein P300 to define SEs^29,30^. However, the knowledge about the ability of these and several other factors to define a set of SEs in a particular cell-type and their relative and combinatorial importance remains limited. The master TFs which form the SE domains are largely unknown for most of the cell-types. Current studies demonstrated the possibility of multiple cofactors with important roles in SE formation. Nevertheless, a computational model that integrates various types of data to predict SEs and their constituents (individual enhancers within an SE), and to identify key signatures has not been developed. In addition, the degree to which the sequence-specific features of the constituents themselves explains the differences between SEs and TEs remains unknown.

Herein, to identify key features of SEs and to investigate their relative contribution in the prediction of SEs, we integrated diverse types of publicly available datasets, including ChIP-seq data for histone modifications, chromatin regulators and TFs, DHSs, and genomic data. Using predictive modeling, we found that H3K27ac, Brd4, Cdk8, Cdk9, Med12, p300, and Smad3 with higher predictive importance and significantly correlated with Med1. We implemented and compared six different parametric and non-parametric machine learning models including Random Forest, Support Vector Machine (SVM), *k*-Nearest Neighbor (*k*-NN), Adaptive Boosting (AdaBoost), Naive Bayes (NB), and Decision Tree. We validated these models using 10-fold stratified cross-validation and also by using independent datasets in four human cell-types. We also used publicly available data to define SEs by using Cdk8, Cdk9, and Smad3 in mouse ESC (mESC) and by using Smad3 in pro-B cells. Our comprehensive machine learning based analysis discovered new signatures of SEs and ranked them based on their predictive importance. Our feature ranking and analysis can serve as a resource to further characterize and understand SEs in other cell-types.

## Results

### Chromatin regulators and TFs at the constituents of SEs and TEs

**Figure 1:**
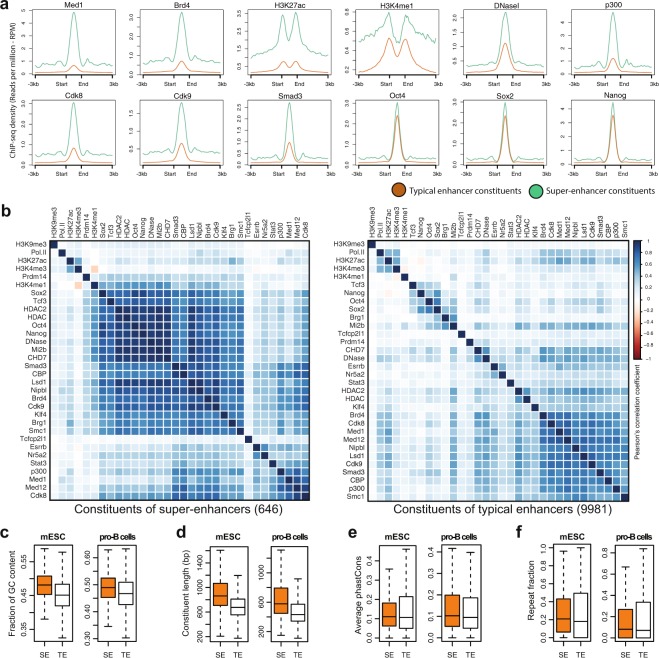
Chromatin regulators, TFs and sequence signatures of the constituents of SEs and TEs. (**a**) Average ChIP-seq profile (RPM) of Med1, Brd4, H3K27ac, H3K4me1, DNaseI, p300, Cdk8, Cdk9, Smad3, Oct4, Sox2 and Nanog at the constituents of SEs and TEs, and their flanking 3 kb regions (**b**) Correlation plot using Pearsons’ correlation coefficient with hierarchical clustering of normalized ChIP-seq signals (rpm/bp) of 32 factors at the constituents of SEs (646) and TEs (9981). More factors were correlated at SEs as compared to TEs, but the factors with similar lineage/functionality were clustered together, for example, active histone modifications (H3K27ac and H3K4me3), the Mediator subunits (Med1, Med12, and Cdk8), and the pluripotency factors of ESC (Oct4, Sox2, Nanog). (**c**) Box plot shows the fraction of GC content, across the constituents of SEs and TEs in mESC and pro-B cells (p-value < 2.2e-16, Wilcoxon rank sum test). (**d**) Constituent enhancers size (bp) in mESC and pro-B cells (p-value < 2.2e-16, Wilcoxon rank sum test). (**e**) Conservation score (phastCons) in mESC (p-value = 0.6285, Wilcoxon rank sum test) and in pro-B cells (p-value < 1.7e-4, Wilcoxon rank sum test). (**f**) Repeat fraction in mESC (p-value = 0.0202, Wilcoxon rank sum test), pro-B cells (p-value = 0.8976, Wilcoxon rank sum test).

Studies have shown that SEs are occupied by various cofactors, chromatin regulators, histone modifications, RNA Pol II, and TFs^17,18^. A thorough understanding of the occupancy of these factors at the constituents of SEs and TEs is not well characterized yet. Therefore, we extensively analyzed 32 publicly available ChIP-seq and DNase-seq datasets to unveil their association with the constituents of SEs and TEs in mESC. We found that most of these factors, which are enriched in SEs, were also highly enriched in the constituents of SEs relative to TEs (Figs 1a and S1). It is expected to observe nearly equal enrichment of Oct4, Sox2, and Nanog at the constituents of SEs and TEs as these constituents are defined using OSN co-bound regions^17^.

Through correlation analysis, we found most of the factors were highly correlated at the constituents of SEs as compared to the constituents TEs (Fig. 1b). Interestingly, features with similar lineage/functionality were clustered together. For example, active histone modifications (H3K27ac and H3K4me3), the Mediator complex subunits (Med1, Med12, and Cdk8), and the pluripotency factors of ESC (Oct4, Sox2, Nanog) were clustered together. The repressive histone modification H3K9me3 which is associated with heterochromatin^31^ was not clustered with any of the other factors both at SEs and TEs. It was particularly interesting to observe that Smad3 clustered with the coactivator protein p300/CBP. Previous studies have shown that p300/CBP interacts with Smad3^32,33^. Taken together, with previous studies this suggests a potential combinatorial interplay between these cofactors at the constituents of SEs and that may explain why SEs are transcriptionally more active and sensitive to perturbation, and occupied by diverse transcriptional apparatus.

### Sequence signatures of SEs and their constituents

SEs differ from TEs in terms of size, ChIP-seq density of various cofactors, TF content, ability to activate transcription, and sensitivity to perturbation^17–19^. But, to what extent the constituents of SEs differ from the constituents of TEs in terms of sequence composition remains unknown. To gain insights, we sought to identify DNA sequence signatures of constituents. We tested GC-content, repeat fraction, size, and sequence conservation scores (phastCons) scores across the constituents of SEs and TEs in mESC and pro-B cells.

By looking at the GC-content, we found that constituents of SEs are significantly more GC-rich than the constituents of TEs (p-value < 2.2e-16, Wilcoxon rank sum test) (Fig. 1c). Previous studies have shown that GC-rich regions have distinct features including frequent TF binding^34^, active conformation^35^, and nucleosome formation^36^. These results suggest that GC richness may have important roles in the formation of SEs and it can be a defining feature to distinguish them from TEs.

It has been shown that enhancers larger than 3 kb tend to be more cell-type specific and are known as stretch enhancers^27^. Hence, we checked the size (bp) of constituents and found that constituents of SEs are significantly larger in size than the constituents of TEs (p-value < 2.2e-16, Wilcoxon rank sum test) (Fig. 1d). This suggests that SEs are a subset of stretch enhancers, which we showed in our recent work^37^.

Next, we looked at the phastCons scores at the constituents of SEs and TEs. It has been shown that enhancers are rarely conserved across mammalian genomes and evolved recently from ancestral DNA exaptation, rather than lineage-specific expansions of repeat elements^38^. We did not find any significant difference in conservation at constituents of SEs and TEs in mESC (p-value = 0.6285, Wilcoxon rank sum test). In contrast, in pro-B cells, the conservation score was significantly higher (p-value < 1.7e-4, Wilcoxon rank sum test) (Fig. 1e), which may suggest that SEs are evolutionarily conserved in differentiated cells. We also looked at the repeat fraction and did not see any significant difference at the constituents of SEs and TEs in pro-B cells (p-value = 0.8976, Wilcoxon rank sum test), but significant in mESC (p-value = 0.0202, Wilcoxon rank sum test) (Fig. 1f). Taken together, these results show roughly the same levels of phastCons and repeat fraction at the constituents.

### Feature-ranking revealed previously known and new features of SEs

**Figure 2:**
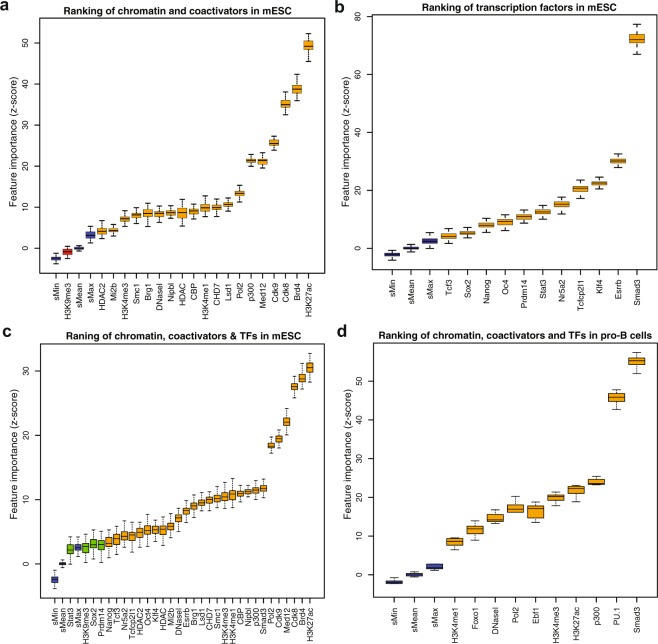
Ranking of features including chromatin regulators and TFs in mESC and pro-B cells. (**a**) Box plot shows the importance of features, including histone modifications, chromatin regulators, coactivators, DNaseI and other features. The feature importance is calculated by using a Random Forest based algorithm, Boruta. The colors represent (Blue = shadow features; Red = negative features; Orange = Important features; Green = Neutral features). (**b**) Box plot shows the feature importance for 11 TFs in mESC. (**c**) Box plot shows the importance of feature after combining the chromatin, coactivators and TFs features in mESC. (**d**) Box plot shows the importance features, including chromatin, coactivators and master TFs in pro-B cells.

With the increasing number of factors identified and associated with SEs, determining their relative importance in defining SEs is critical. Therefore, we ranked chromatin regulators and TFs to find a minimal optimal subset, which can be used to optimally distinguish SEs from TEs. We used a random-forest based algorithm, Boruta^39^, to assess the relevance of each feature by ranking them based on their predictive importance (Fig. 2a) (Methods). As SEs were defined using Med1, to avoid bias we excluded Med1 from feature ranking and also from model training. To further verify, we used an out-of-bag approach to calculate the relative importance of each feature and achieved almost identical results (Figure S2). The feature importance scores computed by both methods were significantly correlated (Pearson’s r = 0.65; p-value = 8.473e-05) with a 95% confidence interval (Figure S3).

Through ranking of chromatin features, we found Brd4, H3K27ac, Cdk8, Cdk9, Med12, and p300 as the six most important factors with Brd4 and H3K27ac as the top two most informative features (Fig. 2a). It was particularly interesting to observe Cdk8 and Cdk9 were ranked as the third and fourth most informative features, respectively. Cdk9, a subunit of the positive transcription elongation factor b (P-TEFb) was found in enhancers and promoters of active genes along with the Mediator coactivator^19^. Cdk8, a subunit of Mediator complex, positively regulates precise steps in the assembly of transcriptional elongation, including the recruitment of P-TEFb and Brd4^40^.

Previous studies have shown that several ESC specific TFs (Sox2, Oct4, Nanog, Esrrb, Klf4, Smad3, Stat3, Tcf3, Nr5a2, Prdm14, and Tcfcp2l1) were enriched in SEs^17,18^. As these TFs are specific for ESC biology, we ranked them separately from other chromatin regulators to find their relative importance in ESC. Interestingly, Smad3 turn out to be the most informative feature among the other TFs including Klf4 and Esrrb which were previously described as key defining features of SEs^17^ (Fig. 2b). It will be particularly interesting to further understand the contribution of Cdk8, Cdk9, and Smad3 in defining and predicting SEs.

We also ranked the chromatin regulators and TFs together and found H3K27ac, Brd4, Cdk8, and Cdk9 as the most informative features, while Smad3 was ranked higher than p300 and ESC specific master TFs (Fig. 2c). To test this in a more differentiated cell-type, we ranked several factors, including H3K27ac, H3K4me1, H3K4me3, p300, Smad3, PU.1, Foxo1, Ebf1, Pol2, and DNaseI in pro-B cells. Interestingly, we found that Smad3 again turn to be the most informative feature followed by Pu.1, p300, and H3K27ac (Fig. 2d). A previous study has shown that Smad3 co-binds with cell-type specific master TFs on a genome-wide scale^41^. Taken together, these results suggest that Smad3 is the most informative feature to distinguish SEs in differentiated cells, but this needs further validation in other differentiated cell-types. Moreover, this analysis provides a ranked list of previously characterized and new features that could be used to define SEs.

### Integrative modeling to predict SEs

Next, to assess the individual and combinatorial predictive power of the top-ranked features, we developed a supervised machine learning workflow by integrating six state-of-the-art machine learning models and several types of features. The detailed workflow of our computational prediction pipeline is illustrated in (Figure S4). To summarize, we used SEs as positive and TEs as negative training sets in mESC. We used various sequence and chromatin data to compute feature values at SEs and TEs (Methods). We then applied various data sampling approaches to balance the datasets and train the six different models individually. We validated the predictive performance of models using 10-fold cross-validation. We also tested the trained models on independent datasets, not used in the training.

Since our training data was highly imbalanced, containing fewer SEs and more TEs, and to avoid overfitting, we applied three sampling strategies (Figure S5A–B). First, we performed under-sampling by randomly selecting a sub-set, similar in size to the minority class. We evaluated the performance by repeating this process 100 times and achieved an average area under the curve (AUC) of 0.90. Second, we used one of the widely-used over-sampling methods, SMOTE^42^, to increase the minority class and achieved an AUC of 0.98. Third, we used a hybrid approach by first applying over-sampling on the minority class and then under-sampling on the majority class. In general, the predictive performance of the model increased after performing data sampling. Using imbalanced data, the model achieved AUC = 0.87 but very poor precision-recall curve (PRC) of 0.57. We achieved an equal AUC = 0.98 after using over-sampling and hybrid-sampling approach but a stable PRC = 0.98, only using hybrid sampling. Therefore, we selected the hybrid-sampling approach for further analysis.

**Figure 3:**
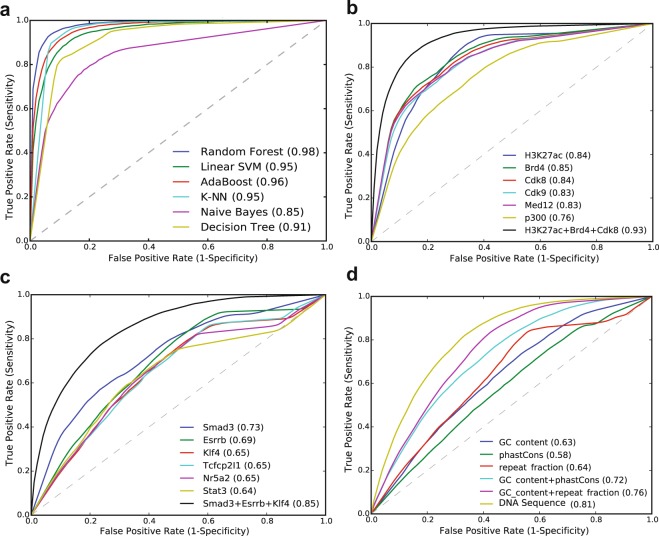
Integrative modeling to compare machine learning methods and predictive power of chromatin, TFs other genomic features. (**a**) ROC plot shows the AUCs for six state-of-the-art machine learning models using all features. (**b**) Predictive power of top-ranked 6 features including Brd4, H3k27ac, Cdk8, Cdk9, Med12 and p300. (**c**) Predictive power of top-ranked 6 TFs including Smad3, Klf4, Esrrb, Stat3, Tcfcp2l1 and Nr5a2. (**d**) ROC plot for sequence-specific features.

We then compared the six supervised machine learning models, including Random Forest, linear SVM, k-NN, AdaBoost, Naive Bayes, and Decision Tree by using all the features. We used chromatin regulators, TFs, and sequence-specific features to train these models individually, and evaluated their performance using 10-fold cross-validation. Using all features, the Random Forest performed the best with an AUC = 0.98, while Naive Bayes performed poorly with an AUC = 0.85 (Figs 3a, S5D). Linear SVM and k-NN performed equally with AUC = 0.95, while Decision Tree achieved AUC = 0.91. We also compared non-linear variants of SVM (Radial basis function (RBF) and Polynomial kernels) with linear SVM and did not observe any bias (Figure S7). We found that AdaBoost performed almost equally, with an AUC = 0.96 compared to the Random Forest which had an AUC = 0.98, but we achieved a stable precision and recall only for the Random Forest. We further compared these models, using individual features as well as different types of features and found feature-type specificities for each model (Figure S6; Tables S1 and S2). For example, the linear SVM performed poorly on DNA sequence-specific features with an AUC = 0.69, while the Random Forest and AdaBoost performed best with an AUC = 0.81. In general, the Random Forest model performed the best across different combinations of features and data sampling approaches. Random Forests are the ensemble and non-parametric models, which run efficiently on large datasets without over-fitting compared to other models (Figures S8 and S9). Hence, we selected the Random Forest model for further analysis of our features due to its performance and flexibility, though any of these models can be used to predict SEs to some extent depending on the type of features used. Taken together, these results suggest that ensemble predictive modeling techniques perform well in general and our comparative analysis of models using various types of features can be used as a guide to select the best model based on the type of features available.

### Predictive ability of top-ranked chromatin and genomic features

We applied our integrative workflow to test the predictive ability of the top-ranked chromatin and genomic features. As expected, the features which were ranked as the most important predictors (Fig. 2a,b), achieved higher AUC and PRC scores. Among the top-ranked chromatin features, including H3K27ac, Brd4, Cdk8, Cdk9, Med12, and p300, we observed Brd4 performed slightly better than H3K27ac, with an AUC = 0.85 and 0.84, respectively. Interestingly, our model achieved an equal AUC = 0.84 for H3K27ac and Cdk8. Furthermore, it achieved an AUC = 0.83 for Cdk9, 0.83 for Med12 and 0.76 for p300 (Fig. 3b). After combining the top three chromatin features (H3K27ac, Brd4, and Cdk8) the model performed well with an AUC = 0.93. This shows that the combination of Brd4, H3K27ac, and Cdk8 is more effective at predicting SEs than either one individually. Among the top-ranked TFs, including Smad3, Esrrb, Klf4, Tcfcp2l1, Nr5f2a, and Stat3, we found that Smad3 achieved the highest predictive power with an AUC = 0.73, followed by Esrrb with an AUC = 0.69 and Klf4 with an AUC = 0.65 (Fig. 3c). The model achieved an AUC = 0.65, 0.65, 0.64 for Tcfcp2l1, Nr5f2a, and Stat3, respectively. After combining the top three ranked TFs, including Smad3, Esrrb and Klf4, the model performed better with an AUC = 0.84 than individual TFs. This shows that the combinatorial information of cofactors is more effective at predicting SEs than the individual features alone. We also noticed that the top-ranked features, including Cdk8, Cdk9, p300, CBP, Smad3, and Brd4 are highly correlated with Med1, at the constituents of SEs and TEs (Fig. 1b). Taken together these results show that the top-ranked individual features can accurately predict SEs, but the prediction performance greatly improves when combining the top three features.

### Predictive ability of DNA sequence signatures

To assess if SEs can be predicted by using DNA sequence information only, we computed DNA sequence-specific features, including conservation scores (phastCons), GC content and repeat fraction, and investigated their individual and combinatorial predictive power. The model achieved an AUC = 0.58 for phastCons, 0.63 for GC content, and 0.64 for repeat fraction (Fig. 3d). By combining the GC content and phastCons the model achieved an AUC = 0.71, and by combining GC content and repeat fraction it achieved an AUC = 0.76. By using all three of the sequence-specific features, the model performed significantly higher with an AUC = 0.81. Next, we used the DNA motifs for the 11 TFs (Oct4, Sox2, Nanog, Esrrb, Klf4, Tcfcp2l1, Prdm14, Nr5a2, Smad3, Stat3, and Tcf3) to train the model. Using only the motif information, the model achieved an AUC = 0.72. We also tested prediction accuracy using a sequence-specific *k*-mer based approach^43^. We achieved an AUC = 0.74 for stitched sequences of SEs and TEs, and an AUC = 0.75 for the constituents of SEs and TEs (Figure S5F,G). Taken together, these results show that only the DNA sequence features could be enough to predict SEs where high throughput sequencing data is not available.

### Combinatorial predictive ability of chromatin, genomic and sequence features

**Figure 4:**
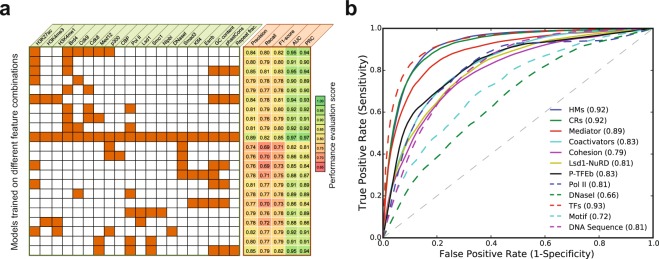
Combinatorial predictive ability of chromatin and sequence signatures. (**a**) Predictive power of models trained on different combinations of features based on their functional importance. (**b**) ROC plot shows the AUCs of features grouped based on their type.

Previous studies have shown that a combinatorial interplay between multiple histone modifications, TFs, and DNA motifs, and this can be functionally informative^44,45^. Above, we noticed significant correlation among many chromatin regulators and TFs at the constituents of SEs. This suggests the existence of a combinatorial relationship betwe’en these factors, which might dictate an accurate explanation for their role in SEs formation. This combinatorial information could also be more predictive than the individual information of each factor. We, therefore, investigated the combinatorial predictive power of chromatin, TFs, and sequence-specific features. We tested 22 different combinations of these features and reported precision, recall, F1-score, AUC, and PRC (Fig. 4a). By training the model with the top six chromatin features, including Brd4, H3K27ac, Cdk8, Cdk9, Med12, and p300, it achieved an AUC = 0.95. By training the model with the top two features (Brd4 and H3K27ac), it achieved an AUC = 0.91. Further adding the sequence-specific features, the predictive ability of the model greatly increased with an AUC = 0.95. By combining histone modifications, including H3K27ac, H3K4me1, and H3K4me3, with sequence-specific features, it achieved an AUC = 0.94. When using known enhancer features, including H3K4me1, H3K27ac, p300, and DNaseI, it achieved an AUC = 0.92. It is well known that the Mediator forms a complex with Cohesin to create cell-type specific DNA loops, and it facilitates enhancer-bound TFs to recruit RNA Pol II to the promoters of the target genes^14,15^. Therefore, when we tested the combinatorial predictive power of the Mediator sub-unit Med12, Cohesin sub-unit Smc1, and RNA Pol II, the model achieved an AUC = 0.91. Adding the sequence-specific features, the model performance considerably improved to an AUC = 0.95.

Furthermore, we tested the predictive ability of the features that were grouped based on their type and functionality, and found that TFs, histone modifications and chromatin regulators have higher predictive power with an AUC = 0.93, 0.92 and 0.92, respectively (Figs 4b and S6; Table S2). For the models trained on the Mediator complex, Cohesin, coactivators and Pol II data, we achieved an AUC = 0.89, 0.79, 0.83 and 0.81, respectively (Fig. 4b). It was particularly interesting to see that a model trained on genomic features, including GC content, phastCons, and repeat fraction, achieved AUC = 0.81. Taken together, our analysis shows that the combinatorial information greatly increased the predictive power of the models. Moreover, the sequence-specific features alone are reliable predictors, abut adding other features enhances their predictive power.

### Model validation using independent datasets

The above tested models performed well on mESC data using 10-fold cross-validation as a validation strategy. To further validate, we used independent datasets, which were not used during the model training. We used publicly available data in four human tumor cell-types, including B-cell lymphoma (P493-6), multiple myeloma (MM1.S), small cell lung carcinoma (H2171) and glioblastoma (U87). We chose these cell-types because ChIP-seq data for MED1 and the two top-ranked chromatin features, BRD4 and H3K27ac, were publicly available (Table S4). Initially, we used H3K27ac ChIP-seq peaks, 2 kb upstream and downstream from the transcription start site (TSS), to define constituent enhancers and ranked them based on the MED1 ChIP-seq signal to define SEs as described in^17–19^.

**Figure 5:**
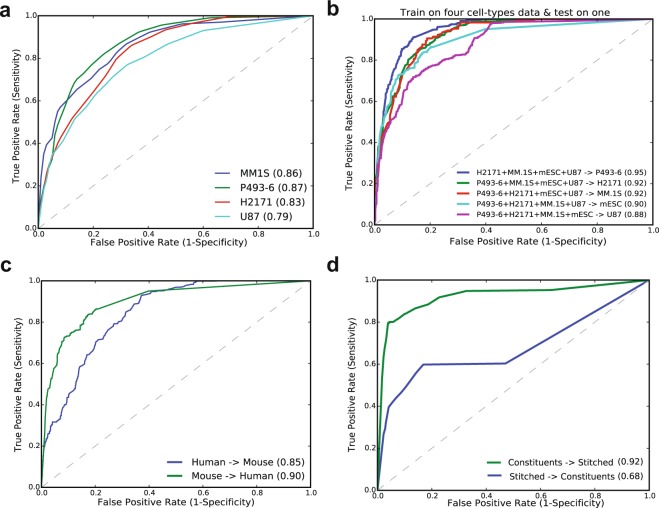
Model validation using independent datasets. (**a**) Model validation using independent data in four human cell-types. (**b**) ROC plot for model training on four cell-type data and testing on one cell-type data. (**c**) ROC plot for model trained on one genome and test on another. (**d**) Predicting stitched SEs using model trained on constituents.

We then trained the model on mESC data and applied it on each of the four human cell-type data and achieved an AUC = 0.92 for P493-6, 0.90 for MM1.S, and 0.86 for U87 cells (Fig. 5a). We also checked the classification accuracy after combining five cell-types data by training the model on four cell-types data and testing it on the remaining cell-type, repeating this for all the combinations (Fig. 5b). The classification measures, including precision, recall, F1-score, and AUC for each tested cell-type after training the model on the other four cell-types are listed in (Table S1). We achieved the highest AUC = 0.95 for the model tested on P493-6 cell-type data and the lowest AUC = 0.88 for the model tested on the U87 cell-type. We next trained the model on one genome data and tested on another. We used four human cell-types (P493-6, Plasma cell, H2171 and U87), and one mouse cell-type (mESC) data. The model was trained on mouse cell-type data and applied on human cell-type data, achieving an AUC = 0.90 (Fig. 5c). Next, the model was trained on human cell-type data and applied on mouse cell-type data, achieving an AUC = 0.85. We also tested whether a model trained on constituent data can predict the stitched regions and vice versa. Training on H3K27ac data, we accurately predicted the stitched SEs with an AUC = 0.92 (Fig. 5d). The same model, when trained on stitched data and applied on constituent data, performed poorly with an AUC = 0.68. Taken together, these results validate the predictive ability of the features, which can be used be used to accurately predict SEs across cell-types and genomes.

### Identification and characterization of SEs by using Cdk8, Cdk9, and Smad3 in mESC

**Figure 6:**
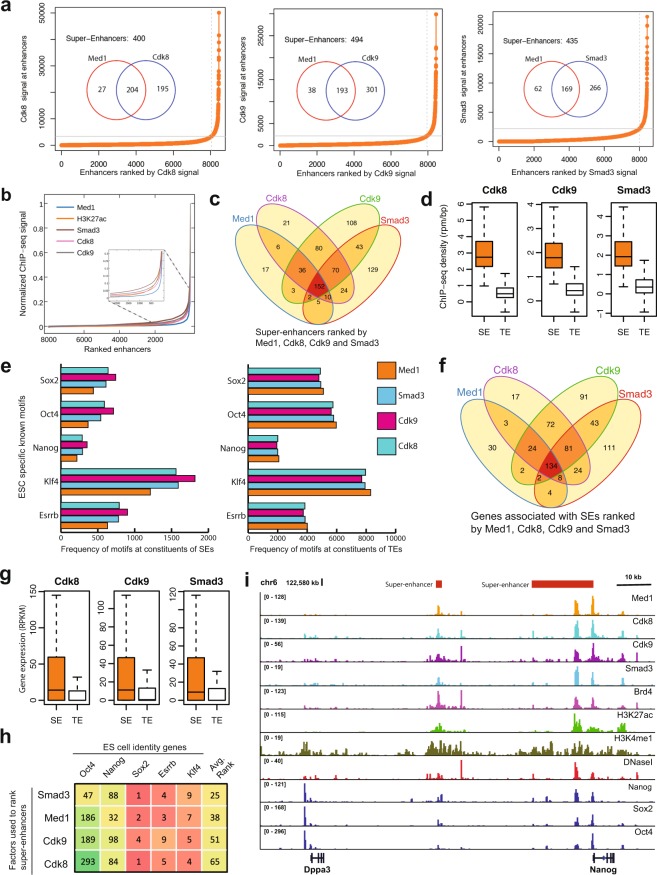
SEs identified by using Cdk8, Cdk9 and Smad3. (**a**) The Hockey-stick plot shows the cut-off used to separate SEs from co-OSN (Oct4, Sox2, and Nanog) regions by using Cdk8, Cdk9 and Smad3. (**b**) The distribution of normalized ChIP-seq signal of Med1, H3K27ac, Cdk8, Cdk9 and Smad3 at mESC enhancers. For each factor, the values were normalized by dividing the ChIP-seq signal at each enhancer by the maximum signal. The rank of enhancer at each factor was measured independently. The figure zoomed at the cut-off so it can be visualized. (**c**) Venn diagram shows the number of SEs overlapped, ranked using Med1, Cdk8, Cdk9 and Smad3. (**d**) Boxplot shows the ChIP-seq density (rpm/bp) in SEs and TEs defined by Cdk8, Cdk9 and Smad3 (p-value < 2.2e-16, Wilcoxon rank sum test). (**e**) Bar-plot shows the frequency of motifs (Oct4, Sox2, Nanog, Klf4 and Essrb) found at the constituents of SEs and TEs defined by Med1, Cdk8, Cdk9 and Smad3. (**f**) Venn diagram of genes associated with SEs identified using Med1, Cdk8, Cdk9 and Smad3. The genes associated to Med1 SEs are downloaded from dbSUPER. (**g**) Boxplot shows the gene expression (RPKM) in SEs and TEs defined by Cdk8, Cdk9 and Smad3. (**h**) Rank of factors based on the rank of SEs associated with of ESC identity genes, including Sox2, Oct4, Nanog, Esrrb and Klf4. The table is sorted based on the average rank. (**i**) ChIP-seq binding profiles of different factors at the TEs and SEs at Dppa3 and Nanog gene locus in mESC.

Through the ranking of chromatin regulators and TFs we found that Cdk8, Cdk9, and Smad3 were important features along with many well-known chromatin signatures of SEs, including H3K27ac, Brd4, Med12, and p300^17–20,29^. However, little is known about the binding of Cdk8, Cdk9, and Smad3 at SEs. To validate the importance of these features further, we first characterized the genome-wide ChIP-seq binding of Cdk8, Cdk9, and Smad3 at SEs identified by using Med1 in mESC and observed that Cdk8, Cdk9, and Smad3 are highly correlated with Med1 and co-occupy SEs genome-wide (Figures S10 and S11). We then investigated the importance of Cdk8, Cdk9, and Smad3 in the formation of SEs and also compared them with the SEs identified by Med1. We used ChIP-seq data and RNA-seq data to identify and characterize SEs by using Cdk8, Cdk9, and Smad3 in mESC. We identified 400, 494 and 435 SEs by using Cdk8, Cdk9, and Smad3, respectively (Fig. 6a) listed in Supplementary Table 1. Furthermore, Cdk8, Cdk9 and Smad3 successfully identified 88%, 84% and 73% of the Med1 SEs (Fig. 6a). After Med1, we can see a clearer distinction of SEs and TEs by using Cdk8 as compared with H3K27ac (Fig. 6b). The majority of the SEs identified using Cdk8, Cdk9, and Smad3 overlaps with the SEs identified using Med1, representing 66% of the SEs (Fig. 6c). The ChIP-seq density at SEs identified using Cdk8, Cdk9, and Smad3 is significantly higher compared with TEs (p-value < 2.2e-16, Wilcoxon rank sum test) (Fig. 6d).

In ESC, the DNA motifs Klf4 and Esrrb were particularly enriched at the constituents of SEs, compared to TEs^17^. Therefore, we tested the frequency of these two motifs at the constituents of SEs and TEs, defined using Cdk8, Cdk9, and Smad3. We found that the frequency of binding motifs Klf4 and Esrrb is significantly higher at constituents of SEs than at TEs (p-value < 2.2e-16, Wilcoxon rank sum test) (Figure S11G). When we compared the frequency of known ESC specific motifs (Oct4, Sox2, Nanog, Esrrb, and Klf4) at the constituents of SEs and TEs defined by Med1, Cdk8, Cdk9, and Smad3 we found a higher frequency at SEs defined by Cdk9, Cdk8 and Smad3 as compared with Med1 (Fig. 6e). The frequency of these motifs was also slightly higher at the TEs identified by Med1 as compared to the ones identified by Cdk8, Cdk9, and Smad3.

As the genes associated with SEs are highly expressed, compared to genes associated with TEs^17–19^. To test this, we associated genes with the SEs and TEs as described in^17,18^. We found that 65% of the Med1 SEs associated genes were also associated with SEs identified by Cdk8, Cdk9, and Smad3 (Fig. 6f). These genes associated with SEs were significantly highly expressed, compared to genes associated with TEs (p-value < 2.2e-16, Wilcoxon rank sum test) (Fig. 6g).

SEs are known to be highly enriched for cell-type specific master TFs, we expect that these TFs should have a higher rank. To test this, we checked the rank of SEs associated with the key cell identity genes, including Oct4, Sox2, Nanog, Esrrb, and Klf4 in ESC selected due to their important roles in the pluripotency and reprogramming of ESC biology^46–48^. We ranked these factors based on the average rank of the SEs associated with these genes (Fig. 6h). The rankings for Med1 SEs were downloaded from Hnisz, D. *et al*.^18^. We found that Smad3 achieved the highest rank followed by Med1, Cdk9, and Cdk8. Smad3 achieved a higher rank for Oct4, compared to the other genes. This might be due to the fact that Smad3 co-binds with the master TFs Oct4 genome-wide in ESC^41^. We found similar ChIP-seq patterns for factors Med1, H3K27ac, Brd4, Cdk8, Cdk9, and Smad3 at SEs regions defined by all three factors (Cdk8, Cdk9, and Smad3) and are associated with cell-type specific genes, including Nanog and Dppa3 (Fig. 6i). Like Med1, the genes associated with SEs ranked by Cdk8, Cdk9, and Smad3 were enriched with cell-type specific GO terms, enforcing the notion that SEs regulate cellular identity genes (Figure S12a). Taken together, our results describe the role of Cdk8, Cdk9, and Smad3 in the definition and formation of SEs.

### Identification and characterization of SEs by using Smad3 in pro-B cells

It was particularly interesting to see Smad3 ranked the most informative feature among the TFs Oct4, Sox2, Nanog, Esrrb, Klf4, Tcfcp2l1, Prdm14, Nr5a2, Stat3 and Tcf3 in mESC (Fig. 2b). In ESC, the highly ranked SEs identified using Smad3 were associated with ESC identity genes Oct4, Sox2, Nanog, Klf4 and Esrrb as compared to the Med1 SEs (Fig. 6h). A previous study showed that Smad3 co-occupies sites with the master TFs Oct4 in mESC, Myod1 in myotubes, and PU.1 in pro-B cells, genome-wide^41^. We also observed that Smad3 as the highest ranked feature in pro-B cells (Fig. 2d).

**Figure 7:**
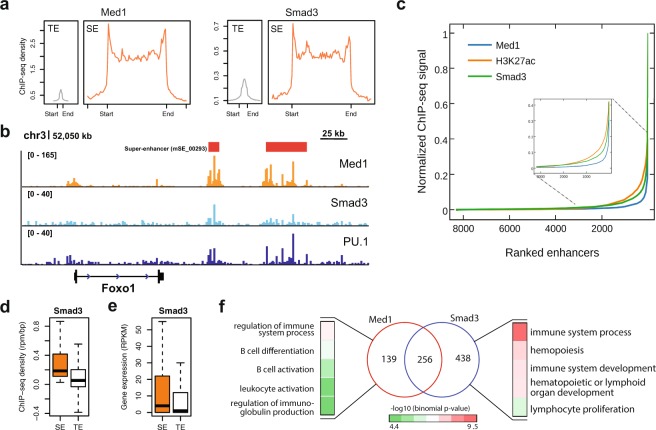
Comparison of SEs ranked using Med1 and Smad3 in pro-B cells. (**a**) Average ChIP-seq density of Med1 and Smad3 across 13,814 TEs and 395 SEs identified using Med1. The flanking region is 3 kb. (**b**) ChIP-seq binding profiles for Med1, Smad3 and PU.1 at the locus of Foxo1 gene. The SE (mSE_00293) is associated with Foxo1 gene. (**c**) The distribution of normalized ChIP-seq signal of Med1, H3K27ac and Smad3 at pro-B enhancers. For each factor, the values were normalized by dividing the ChIP-seq signal at each enhancer by the maximum signal. The rank of enhancer at each factor was measured independently. The figure zoomed at the cut-off so it can be visualized. (**d**) Box-plot shows the Smad3 ChIP-seq density (rpm/bp) at SEs and TEs regions defined using Smad3 in pro-B cells. (**e**) Box plot shows the gene expression (RPKM) for the genes associated with SEs and TEs defined using Smad3 in pro-B cells. (**f**) The Venn diagrams show the overlap of SEs identified using both Med1 and Smad3 in pro-B cells. SE regions identified using Med1 in pro-B were obtained from^17^. Gene Ontology terms (Biological Process) for SEs identified by Med1 only or Smad3 in pro-B cells.

We hypothesized that Smad3 could be used to define SEs where Med1 is not available. As described above, Smad3 could be used to define SEs in mESC. To test this in more differentiated cells, we identified and characterized SEs in pro-B cells by only using Smad3. We compared these SEs with the previously identified SEs using Med1 in pro-B cells^17^. The ChIP-seq density of Smad3 SEs is exceptionally higher compared to TEs (Fig. 7a). The Smad3 TF has strong binding along with Med1 and PU.1 at an SE (mSE_00293) which is associated with the Foxo1 gene (Fig. 7b). By using Smad3, we identified 694 SEs, 65% were also identified by Med1 and the remaining 45% were new (Figure S11f, Supplementary Table 2). Using Smad3, we can see a clearer distinction between the SEs and TEs when compared to H3K27ac (Fig. 7c). The ChIP-seq density at SEs identified using Smad3 is significantly higher, compared to TEs (p-value < 2.2e-16, Wilcoxon rank sum test) (Fig. 7d). The genes associated with the Smad3 defined SEs are significantly expressed, compared to the TEs (p-value < 2.2e-16, Wilcoxon rank sum test) (Fig. 7e). The cell-type specific GO terms for the SEs ranked by Smad3 are highly enriched compared to Med1 (Figure S12b). To test the functional importance of the SEs identified only by Smad3 or by Med1, we performed a GO analysis on genes associated with these subsets of SEs. Interestingly, the SEs identified by Smad3 only are highly enriched for cell-type specific GO terms such as immune cell development and immune system development (Fig. 7f). In contrast, the SEs identified by Med1 but not by Smad3 showed a lower enrichment for cell-type specific GO terms. These results show the importance of Smad3 in SE formation and also suggests that the current standards to define SEs are not ideal.

## Discussion

SEs regulate the expression of key genes that are critical for cellular identity. Thus, alterations at these regions can lead to several disorders. Exploring these cis-regulatory elements and their key signatures is central to uncover the molecular mechanisms and the transcriptional apparatus that orchestrates the cell-type specific gene regulation in development and disease.

In this study, we presented a systematic approach to rank and access the importance of different features of SEs. We investigated histone modifications, chromatin regulators, TFs, DHSs, and DNA sequence motifs in mESC. We also analyzed sequence-specific features including GC content, sequence conservation score and repeat fraction in mESC and pro-B cells. We found new features including Cdk8, Cdk9, Smad3, and GC content as alternate defining features of SEs along with many known features. These features can be used to distinguish SEs from TEs. Further, we developed a supervised machine-learning workflow to assess the combinatorial and individual predictive ability of these features, and subsequently to predict SEs.

Through integrative modeling of chromatin features, we found that Brd4, H3K27ac, Cdk8, Cdk9, Med12, and p300 were the top six features and by assessing their individual predictive powers we achieved higher AUC for higher ranked features. Previous studies showed the importance of these highly ranked features and their role in transcriptional regulation. Through our ranking of features, H3K27ac achieved higher ranking as compared to other histone modifications. H3K27ac is known as a mark to differentiate active enhancers from poised enhancers^11^ suggesting that SEs are clusters of active enhancers. The Mediator sub-units Med1 and Med12 have been known as master coordinators of cell lineage and development^17,49^. Bromodomain-containing protein 4 (Brd4), a member of the BET protein family which functions as an epigenetic reader and transcriptional regulator that binds acetylated lysines in histones^50^, was ranked as the second highest important feature. Brd4 has been associated with anti-pause enhancers (A-PEs) which regulate the RNA Pol II promoter-proximal pause release^51,52^. Brd4 regulates the positive transcription elongation factor b (P-TEFb) to allow RNA Pol II phosphorylation and the subsequent elongation of target genes^52,53^. In ESC it specifically governs the transcriptional elongation by occupying SEs and by recruiting Mediator and Cyclin dependent kinase 9 (Cdk9) to these SEs^54^. Cdk9, a sub-unit of P-TEFb, has been found at enhancers and promoters of active genes along with the Mediator coactivator^19^. Cyclin-dependent kinase 8 (Cdk8), a subunit of Mediator complex, positively regulates precise steps in the assembly of transcriptional elongation, including the recruitment of P-TEFb and BRD4^40^. Another recent study has demonstrated that Cdk8 regulates the key genes associated with SEs in acute myeloid leukaemia (AML) cells^55^. We also showed that Cdk8 and Cdk9 can define known and new SEs in mESC, which further validates their importance in the SE formation and cell identity.

Through integrative modeling of TFs, we found that Smad3, Esrrb, Klf4, Tcfcp2l1, Nr5f2a, and Stat3 were the top-ranked features and by assessing their individual predictive powers we achieved higher AUC for higher ranked features. It was particularly interesting to see that Smad3 was ranked as the best feature among the TFs including Esrrb and Klf4. It is known that Smad3 is a target of the TGF-β signaling pathway, and studies have shown that Smad3 is recruited to enhancers formed by master TFs^41^. We found a significant correlation between Smad3 and coactivators p300/CBP at SEs as previous studies have shown that p300/CBP interacts with Smad3^32,33^. The evidence for the enrichment of Smad3 at SEs shows how the transforming growth factor beta (TGF-β) signaling pathway can converge on key genes that control ES cell identity. A recent study validates these results by showing that SEs provide a platform for signaling pathways, including TGF-β, to regulate genes that control cell identity during development and tumorigenesis^56^. To further validate, we identified and characterized SEs using Smad3 in mESC and pro-B cells. Our analysis through the integration of ChIP-seq and RNA-seq data suggested the importance of Smad3 in the SE formation and cell identity.

By investigating sequence features, we found that the constituents of SEs were significantly GC-rich. The GC-richness of a genomic regions is associated with several distinctive features that can affect the *cis*-regulatory potential of a sequence^34,35^. GC-rich and AT-rich chromatin domains are marked by distinct patterns of histone modifications. GC-rich chromatin domains tend to occur in a more active conformation and histone deacetylase activity represses this propensity throughout the genome^35^. Also, the GC content and nucleosome occupancy are positively correlated^34^, and GC-rich sequences promote nucleosome formation^36^. TFs tend to bind GC-rich regions in the genome, regardless of the distance and orientation^34^. This suggests that there is a role for the GC content in the formation of SEs, which control the cell-type specific gene expression.

The enhancer activity is due to a cooperative and synergistic interplay of different coactivators and TFs^57^. A recent study showed that multiple enhancer variants cooperatively contribute to alter the expression of their gene targets^58^. Nevertheless, it is not well understood whether constituents of SEs work synergistically or additively. The constituents of SEs make frequent physical contacts with each other^59^ and extensive cooperative binding of TFs have been found at SEs^60^. A study in ESC showed the functional importance of SE constituents^56^. Furthermore, two recent studies have suggested additive and functional hierarchy among the constituents of α-globin and Wap SE locus, respectively^61,62^. In contrast, a different study argues that it is still needs to be determined whether the constituents of a SE function synergistically or additively^63^. Through computational modeling and correlation analysis, we noticed a combinatorial relationship between chromatin regulators and TFs at the constituents of SEs. This advances our current understanding on the determinants of SEs and leads us to hypothesize that these combinatorial patterns may be involved in mediating SEs. Moreover, the significant correlation of many cofactors at the constituents of SEs may suggest cooperative and synergistic interactions, but it requires additional experiments to validate. In summary, our systematic analysis of the SEs features and their rankings can be further used as a platform to define and understand the biology of SEs in other cell-types.

## Methods

### Data description

We downloaded 32 publicly available ChIP-seq and DNase-seq datasets in mESC from Gene Expression Ominibus. These include four histone modifications (H3K27ac, H3K4me1, H3K4me3, and H3K9me3), DNaseI, RNA Pol II, transcriptional co-activating proteins (p300, CBP), P-TFEb subunit (Cdk9), sub-units of Mediator complex (Med1, Med12, Cdk8), other chromatin regulators (Brg1, Brd4, Chd7), Cohesin (Smc1, Nipbl), subunits of Lsd1-NuRD complex (Lsd1, Mi2b), and 11 TFs (Oct4, Sox2, Nanog, Esrrb, Klf4, Tcfcp2l1, Prdm14, Nr5a2, Smad3, Stat3, and Tcf3) (Table S4).

To validate the model using independent data, we downloaded ChIP-seq datasets for MED1, BRD4, and H3K27ac in four human tumor cell-types, including B-cell lymphoma (P493-6), Multiple myeloma (MM1.S), Small cell lung carcinoma (H2171) and Glioblastoma (U87), which were not used in the training of the model. The ChIP-seq datasets used to validate the model are listed in (Table S5). To perform features ranking in pro-B cells, we used ChIP-seq data for Med1, PU.1, Foxo1, Smad3, Ebf1, p300, H3K27ac, H3K4me1, H3K4me3 and Pol2 and also DNase-seq (Table S6).

We also obtained RNA-seq based gene expression data (RPKM) from^64^ and^17^ for mESC and pro-B cells, respectively. We downloaded SE regions in mESC and pro-B cells identified using Med1 ChIP-seq occupancy from dbSUPER, which were identified using similar approach^65^. We also used other genomic features including GC content, conservation score (phastCons) and repeat fraction downloaded from the UCSC table browser^66^.

### Data analysis and feature extraction

Initially, ChIP-seq reads were aligned to mouse genome version mm9 using bowtie^67^ (v0.12.9) with the parameters (-k = 1;-m = 1;-n = 2;-e = 70;–best). We calculated read densities for the ChIP-seq datasets at the constituents of SEs (646) and TEs (9981) and normalized them as described in^17,68^. Briefly, for each constituent region, reads were extended by 200 bp and the density of reads per base pair was calculated using bamToGFF (https://github.com/BradnerLab/pipeline). Next, these densities were normalized in units of reads per million mapped reads per base pair (rpm/bp) with background subtraction. We used a similar approach for DNase-seq data but without background subtraction.

The data for model validation was aligned to the human genome version hg19 as described above. We used MACS (Model-based Analysis of ChIP-Seq)^69^ (v1.4.2) to perform the peak calling and to find ChIP-seq-enriched regions over the background. We used a p-value (10^−9^) as the enrichment threshold. To generate wiggle files, we used MACS with parameter -w -S–space = 50.

For DNA sequence motif data, we collected DNA binding motif information (PWM) from the transfac professional database version 2014^70^ for all the 11 TFs (Fig. 2a). We computed the binding affinity score for the constituents of SE and TE sequences using the TF Affinity Prediction (TRAP)^71^ using individual TF’s position weight matrix.

### Data sampling

There are two commonly used data sampling approaches, oversampling and under-sampling. The data used in this analyses was highly imbalanced. To overcome this, we used a hybrid approach by oversampling to increase the size of the minority class and under-sampling to reduce the size of the majority class. We used the Weka implementation of SMOTE^42^ to perform oversampling with the following parameter settings: nearest neighbours = 5, random seed = 1 and oversampling percentage = 500. Under-sampling was achieved by randomly selecting a subset of the same size as the size of the minority class.

### Feature ranking

To find an optimal feature subset, we first used Boruta algorithm^39^ to rank features. Briefly, it finds important features by measuring the relevance of each original feature with respect to a reference attribute using Random Forest. Second, we used Random Forest out-of-bag approach to calculate the relative importance of each feature. Briefly, this approach takes one feature out and measures its relative importance and contribution to the model. The reason behind to use two approaches to rank features was to see the consistency of feature ranking. To test the interpretability of the feature selection approaches, we computed the Pearson correlation between the features importance scores and observed that the feature scores are significantly correlated (Pearson’s r = 0.65; p-value = 8.473e-05) with 95% confidence interval (Figure S3).

### Training data

We downloaded 10627 loci of constituents of SEs and TEs in mESC defined based on Med1 ChIP-seq signal^17^. Among these 646 were constituents of SEs and 9981 were TEs. The median size of enhancer constituents is 703 bp and SE constituents are 862 bp. After performing hybrid-sampling we have 10,336 instances of data and among these 50% (5,168) are constituents of SEs, and 50% (5,168) are constituents of TEs. We considered constituents of SEs as positive class and constituents of TEs as a negative class. In total, we have 45 features, including 20 chromatin features, 11 TFs, 11 DNA motifs, and three sequence-specific features. We excluded Med1 from our training data because SEs were defined based on Med1 ChIP-seq signal^17^. Not surprisingly, we achieved best classification results by using Med1 as a feature.

### Prediction models

We investigated six state-of-art supervised machine learning models including Random Forest^72^, SVM^73^, k-NN^74^, AdaBoost^75^, Decision Tree, and Naïve Bayes.

Random Forests are ensemble and non-parametric models that fit a number of decision tree classifiers on various sub-samples of the dataset and use averaging to run efficiently on large datasets without overfitting. In computational biology, Random Forests are popular due to their performance and flexibility. The major drawback is their lack of reproducibility, as the initialization of the forest is random.

The SVM model transforms the features into a higher dimension by using kernel functions to find an optimal separating hyperplane between two classes. This is achieved by maximizing the margin between the classes. In this study, we used LibSVM^76^ with a linear kernel and regularization parameter C = 1.0, and also compared non-linear variants of SVM (radial basis function (RBF) and Polynomial kernels). A drawback of the SVMs is that they are computationally intensive and sensitive to noisy data.

The *K*-Nearest Neighbor (k-NN) is a non-parametric method, where *k* is the number of nearest training examples in the feature space. A class label is assigned based on a majority vote of its *k* nearest neighbors. The value of *k* is always a positive integer and if *k* = 1 then the class of the single nearest neighbor is assigned.

Adaptive Boosting (AdaBoost) is an ensemble algorithm with meta-estimators, which initially fits on a sequence of weak learners with repeatedly modified versions of the data. To make a final prediction, all the predictions from the weak learners are combined through a weighted majority vote.

Decision Trees are a non-parametric model that predicts the value of a target variable by learning simple decision rules inferred from the data features.

Naïve Bayes (NB) is a simple probabilistic classifier based on the Bayes theorem, which considers all features to be independent of the probability of a class label. In this study, we used the Gaussian Naive Bayes, which assumes the likelihood of the features to follow a Gaussian distribution.

For all the analysis, we used scikit-learn (version 0.14.1), a Python library for machine learning^77^. Model parameters were optimized using scikit-learn’s GridSearch function with cross-validation. The AUC scores reported in this manuscript are based on the Random Forest model, unless stated otherwise. We calculated an out-of-bag error to find the optimal number of trees (n = 20) to use for Random Forest (Fig. S5C). For other models, we used default parameters set in the scikit-learn library.

### K-mer based prediction

We used a sequence-specific enhancer prediction method (Kmer-SVM)^43^ to classify constituents of TEs and SEs. We used the default settings with *k*-mer size = 5, spectrum kernel, regularization parameter (C) = 1.0. We used 5-fold cross-validation for model validation.

### Performance evaluation

We used 10-fold stratified cross-validation (CV) to validate models, which makes the folds by preserving the percentage of samples for each class. Stratified CV is generally considered a better scheme than standard CV in terms of bias and variance^78^. To evaluate the performance of the models, we reported precision, recall, F1-score, area under the ROC curve (AUC) and the precision-recall curve (PRC). The receiver-operating characteristic (ROC) is a graphical representation of the true positive rate (sensitivity) v/s false positive rate (1-sensitivity). The true positive rate is also known as sensitivity or recall. The false positive rate is also known as (1-sensitivity). The F1 score is an accuracy measure, which considers both the precision and the recall of the test to compute the score. We also tested if the increase in model AUC is statistically significant by using permutation test (1000 runs) (Figure S13).

### Identification of constituent enhancers and SEs

We used 10,227 co-bound regions of Oct4, Sox2, and Nanog in ESC and 13,814 regions of master TF PU.1 in murine progenitor B (pro-B) cells as constituent enhancers, which were obtained from^17^. These constituent enhancers were then stitched together within 12.5 kb and +/−2 kb away from TSS. These stitched regions were ranked based on the ChIP-seq signal by using the ROSE software to define SEs^17^. The SEs data downloaded from dbSUPER^65^ was also generated using the same pipeline.

### Assigning genes to SEs and TEs

We assigned genes to SEs and TEs using a proximity rule as described in^17,18^. It is known that enhancers tend to loop and communicate with target genes^7^, and most of these enhancer-promoter interactions occur within a distance of ~50 kb^79^. This approach identified a large proportion of true enhancer/promoter interactions in ESC^80^. Hence, we assigned all transcriptionally active genes to SEs and TEs within a 50kb window.

### Visualization and statistical analysis

We generated box plots using R programming language by extended the whiskers to 1.5x the interquartile range. The P-values were calculated based on the Wilcoxon signed-rank test for box plots, by using wilcox.test function in R. We used ngs.plot^81^, to generate heat maps and normalized binding profiles at the constituents of SEs and TEs and their flanking 3 kb regions.

### Source code availability

To foster the reproducible research, we developed our analysis pipeline as an open-source Python package with a command line interface and made it freely available for academic use at https://github.com/asntech/improse. A detailed documentation can be found at http://improsedoc.readthedocs.io/.

## Supplementary information

Supplementary Materials

Supplementary Table 1

Supplementary Table 2

## Data Availability

All the accession numbers for the publicly available data used during this study are included in the Supplementary Information Tables S3–S6.
