# Supplementary material for: Integrative modeling reveals key chromatin and sequence signatures predicting super-enhancers
Source: Sci Rep. 2019 Feb 27;9:2877. doi: 10.1038/s41598-019-38979-9 (PMC6393462; doi:10.1038/s41598-019-38979-9)
Supplement: Supplementary file 1 — Supplementary Materials [file 41598_2019_38979_MOESM1_ESM.pdf]

Integrative modeling reveals key chromatin and sequence signatures predicting super-enhancers

Aziz Khan[1,2,*] and Xuegong Zhang[1,3,*]

[1] MOE Key Laboratory of Bioinformatics, Bioinformatics Division and Center for Synthetic and Systems Biology, TNLIST/Department of Automation, Tsinghua University, Beijing, 100084, China

[2] Centre for Molecular Medicine Norway (NCMM), Nordic EMBL Partnership, University of Oslo, 0349 Oslo, Norway

[3] School of Life Sciences, Tsinghua University, Beijing, 100084, China

Note: This document contains Supplementary Figures S1–S13 and Tables S1-S3. Also, the public dataset used in this study in Supplementary Table S3-S6.

Supplementary figures

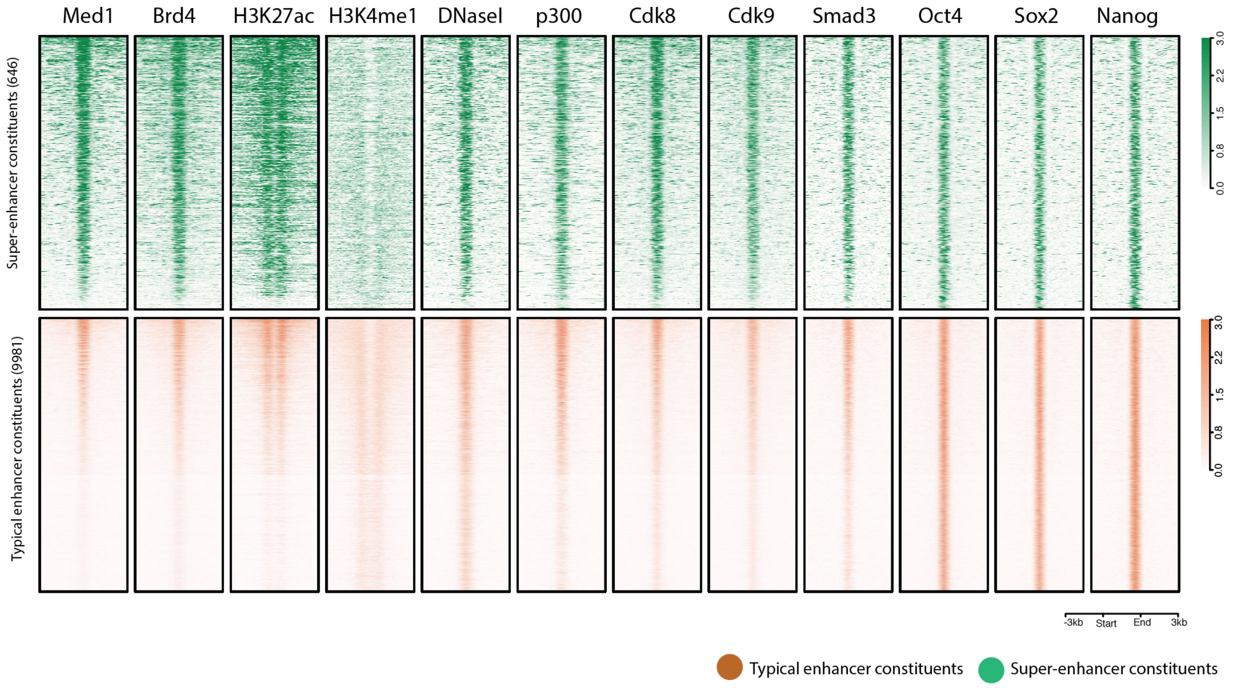

Figure S1. Heatmap of Med1, Brd4, H3k27ac, H3K4me1, DNaseI, p300, Cdk8, Cdk9, Smad3, Oct4, Sox2 and Nanog at the constituents of super-enhancers and typical enhancers and their flanking 3kb regions.

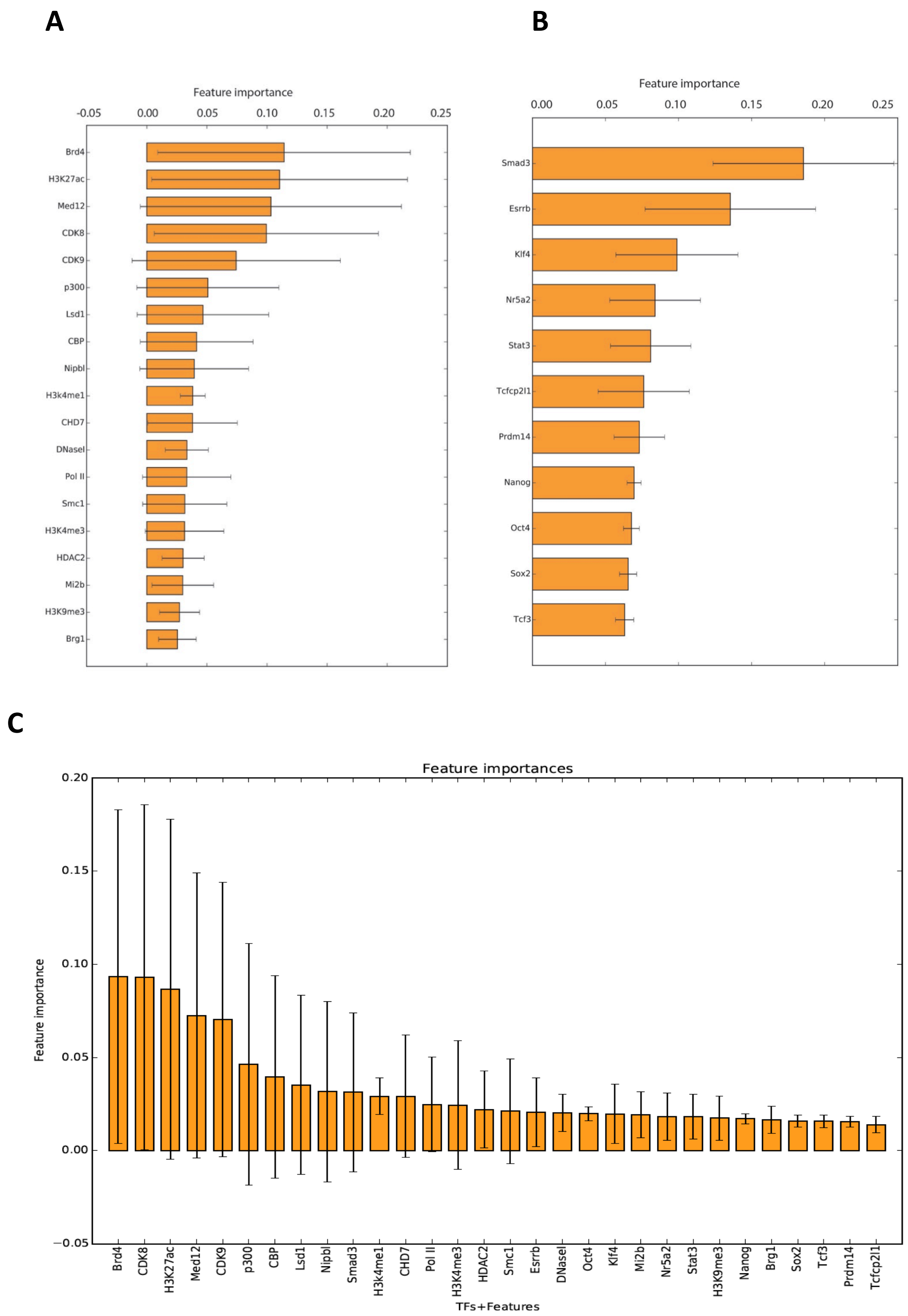

Figure S2: Ranking of features by using Random Forest's out-of-bag approach. (A) Ranking of histone modification, chromatin regulators and coactivators (B) Ranking of transcription factors. (C) Ranking of factors together.

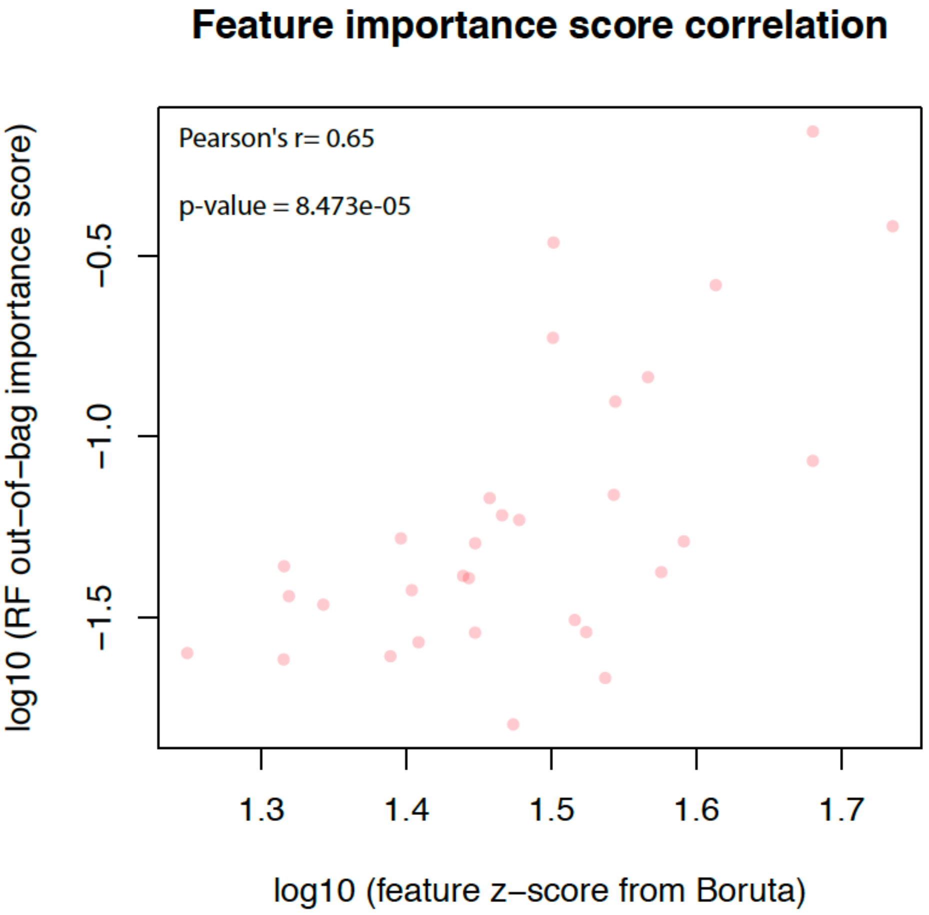

Figure S3. Correlation between the feature importance scores obtained using Boruta and Random Forest out-of-bag approach.

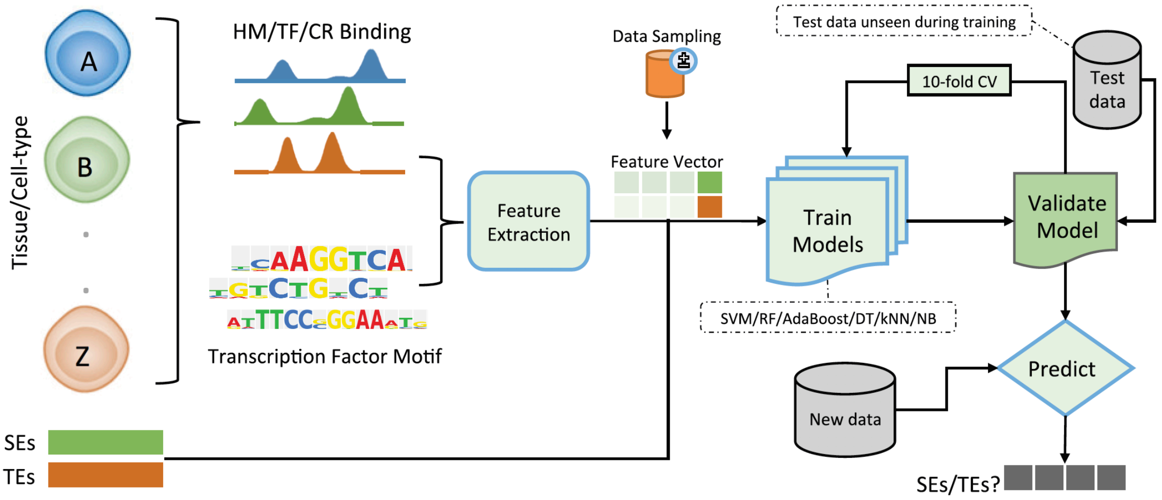

Figure S4: A detailed workflow of our computational prediction and feature analysis pipeline

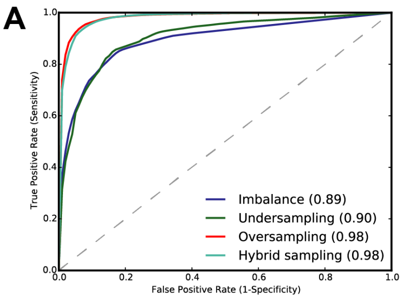

B

Random Forest with 10-fold CV	Precision	Recall	F1.score	AUC	PRC
Hybrid sampling	0.91	0.82	0.86	0.98	0.98
Oversampling (SMOTE)	0.88	0.78	0.83	0.98	0.97
Random Undersampling	0.82	0.72	0.77	0.9	0.91
Imbalance data	0.64	0.42	0.51	0.89	0.57

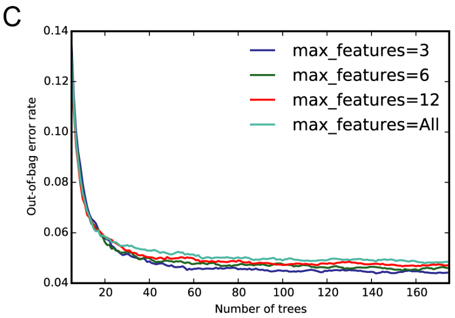

D

	Precision	Recall	F1-score	AUC	PRC
Random Forest	0.91	0.81	0.86	0.98	0.98
AdaBoost	0.90	0.65	0.76	0.96	0.96
Linear SVM	0.83	0.74	0.78	0.95	0.95
k-NN	0.85	0.78	0.81	0.95	0.96
Decision Tree	0.78	0.82	0.80	0.91	0.92
Naive Bayes	0.84	0.69	0.76	0.85	0.87

E

Feature combinations / Performance assesment

HMs	CRs	Mediator	Coactivators	Cohesion	Lsd1-NuRD	DNaseI	Pol II	TFs	GC content	phastCons	Precision	Recall	Fscore	PRC	AUC
✔	✔	✔	✔	✔	✔	✔	✔	✔	✔	✔	0.85	0.78	0.81	0.95	0.95
✔	✔										0.81	0.81	0.81	0.94	0.94
	✔	✔		✔					✔	✔	0.81	0.78	0.8	0.93	0.93
✔						✔		✔			0.83	0.81	0.82	0.94	0.93
			✔		✔		✔				0.78	0.77	0.77	0.9	0.9
✔								✔	✔	✔	0.82	0.81	0.82	0.94	0.93
		✔	✔			✔					0.81	0.73	0.77	0.91	0.92
				✔			✔				0.8	0.7	0.75	0.89	0.88
		✔				✔			✔	✔	0.81	0.76	0.78	0.92	0.92
	✔		✔			✔	✔				0.81	0.78	0.8	0.93	0.93
				✔	✔		✔				0.77	0.78	0.78	0.9	0.89
			✔					✔	✔	✔	0.79	0.78	0.79	0.91	0.9

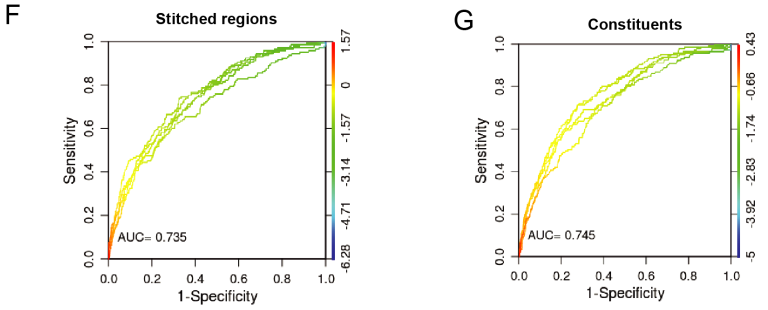

Figure S5: (A) ROC plots data sampling approaches used; imbalance data, undersampling, oversampling and hybrid-sampling. (B) The table shows precision, recall, precision-recall curve (PRC) and area under the curve (AUC) for data sampling approaches. (C) Random Forest out-of-bag error plot to choose optimal number of trees. (D) ROC plots for histone modifications. (E) Combinatorial predictive power of features grouped based on their function. (F-G) ROC plot for k-mer based prediction of SEs constituents as positive set and TEs constituents as negative set using kmer-SVM.

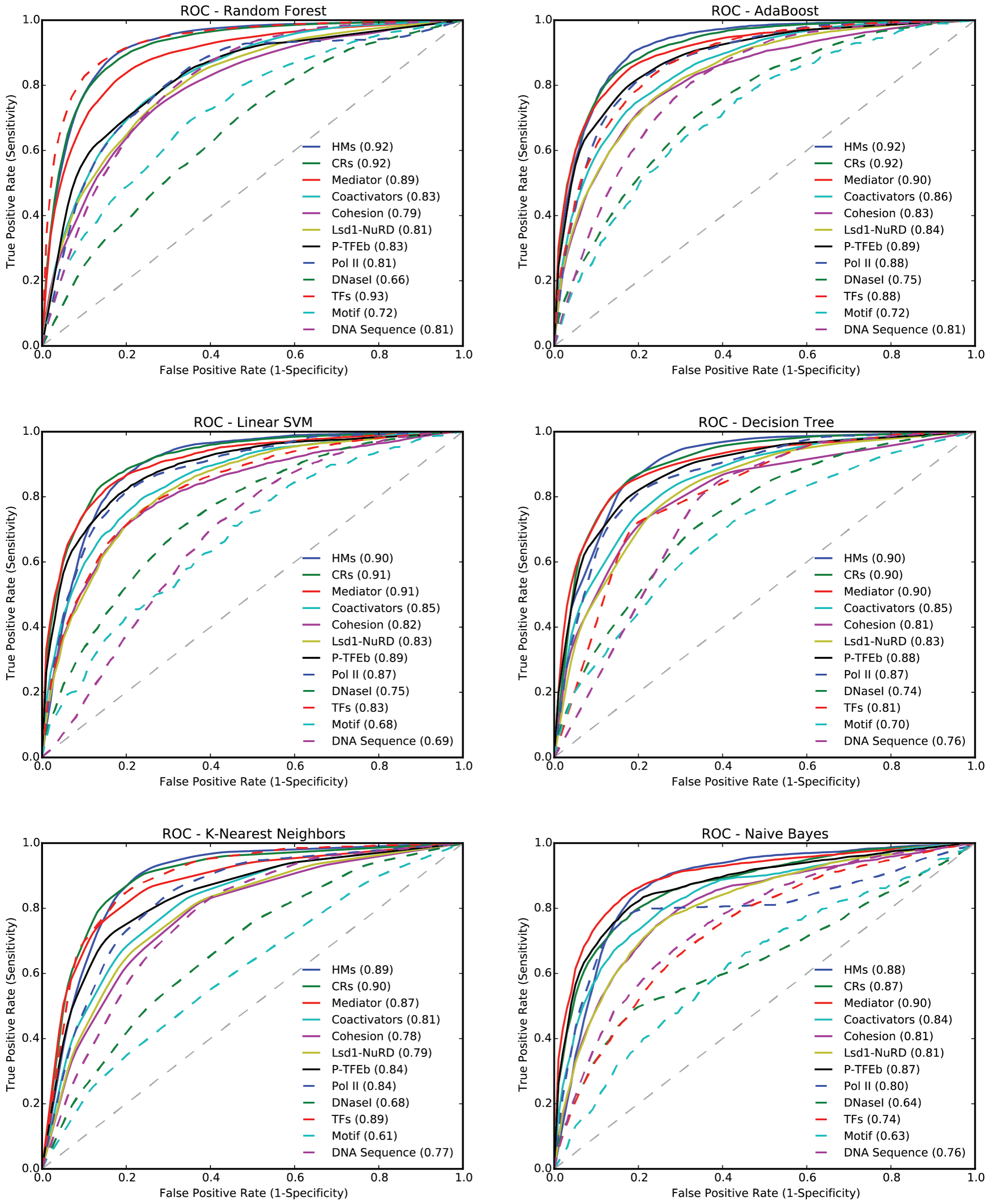

Figure S6: ROC plots comparing six different stat-of-the-art supervised machine learning models, including Random Forest, AdaBoost, linear SVM, Decision Tree, k-NN and Naive Bayes, and different types of features.

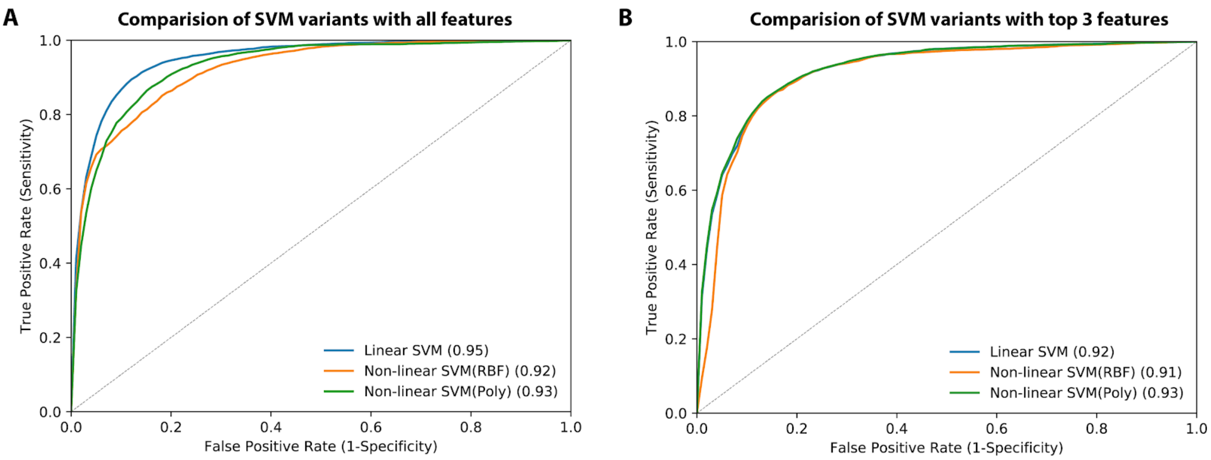

Figure S7: Comparison of linear and non-linear variants of SVM including RBF and Polynomial kernels on full-model (A) and reduced model (B).

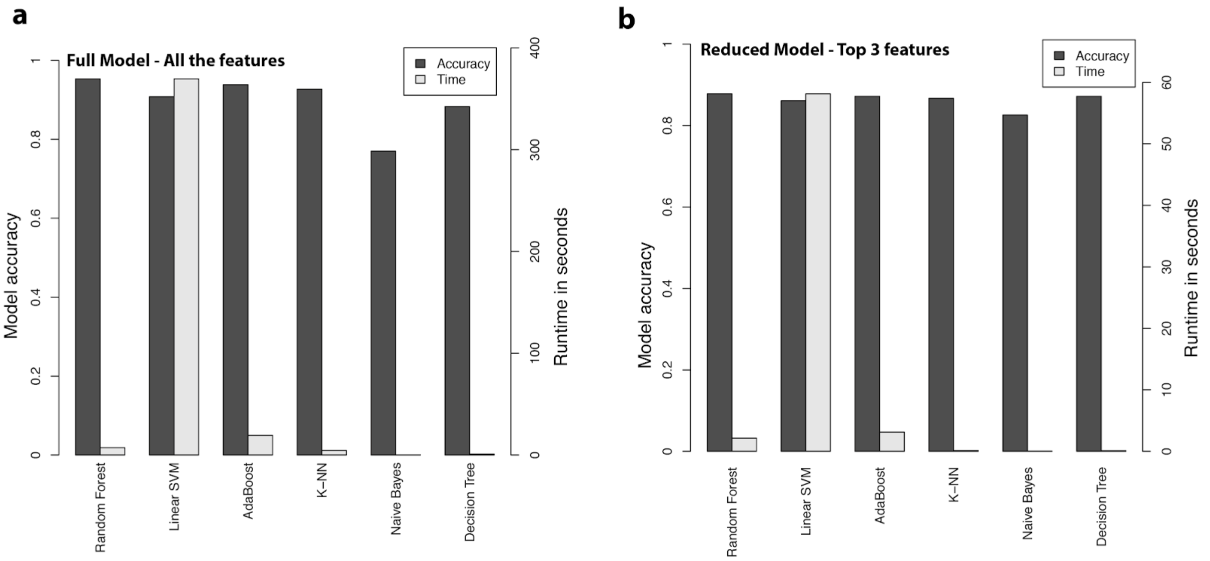

Figure S8: Comparison of runtime (in seconds) and model accuracy for both full-model (all the features) (a) and reduced-model (top 3 features only) (b).

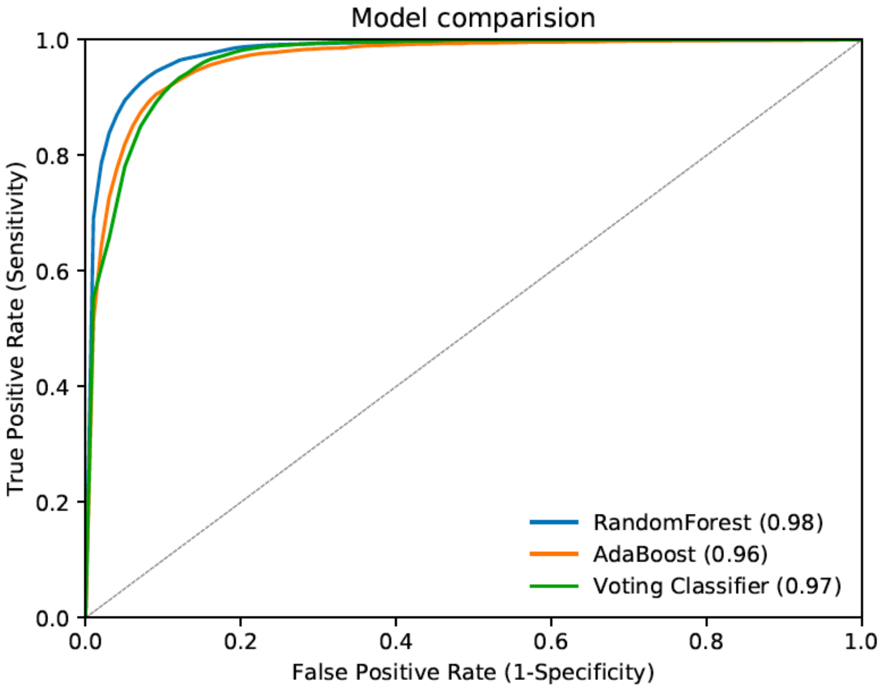

Figure S9: Comparison of ensemble learning models including Random Forest, AdaBoost and Majority Voting. The Majority Voting Classifier uses all the current six classifiers and multi-layer perceptron (MLP) algorithm with backpropagation.

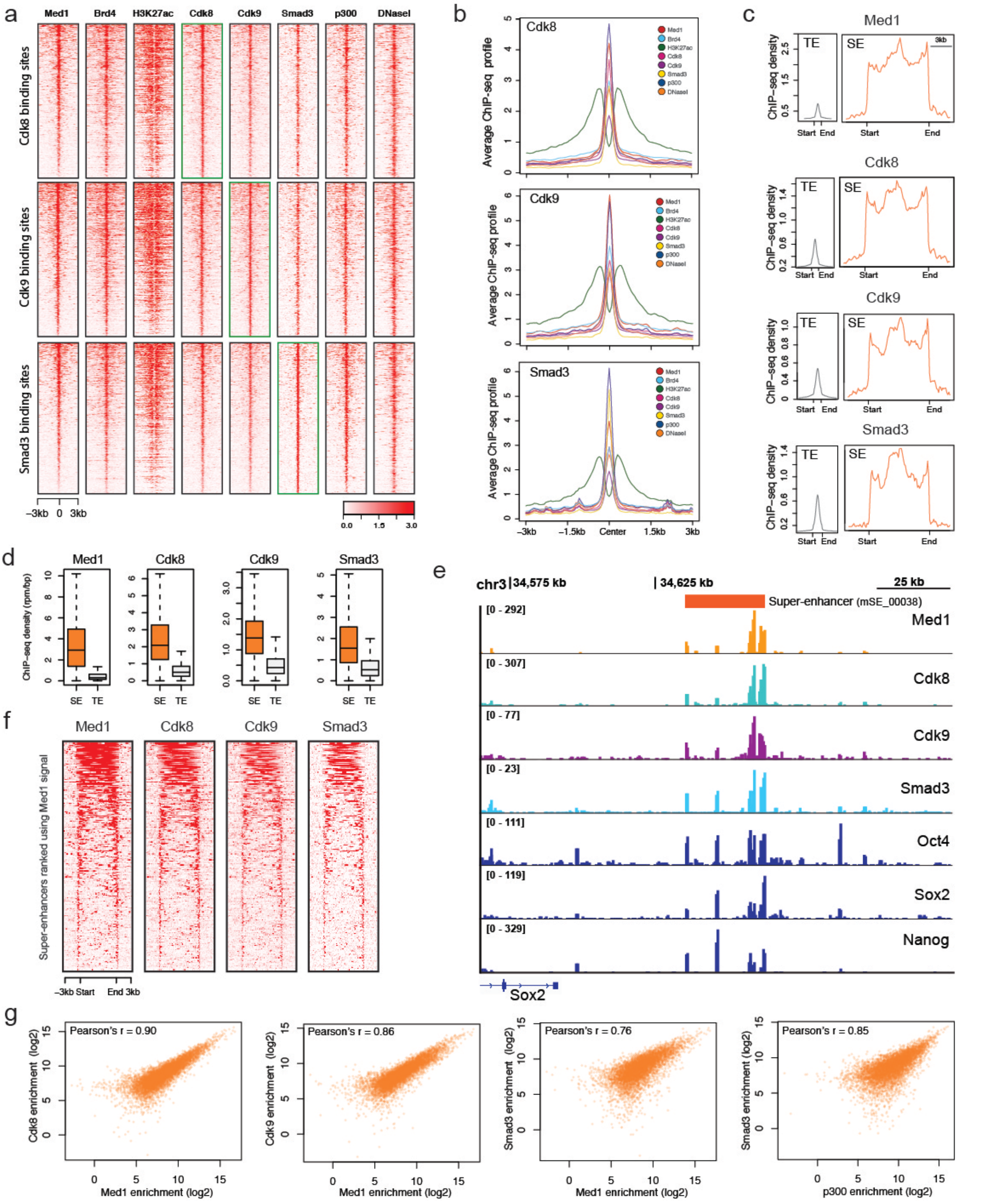

Figure S10. Genome-wide profiles of Cdk8, Cdk9 and Smad3 across super-enhancers and typical enhancers. (a) The heatmap shows the genome-wide ChIP-seq binding profile of Cdk8, Cdk9 and Smad3 across factors including Med1, Brd4, H3K27ac, Crdk8, Cdk9, Smad3, p300 and DNaseI. (b) The average read count profile of factors including Med1, Brd4, H3K27ac, Crdk8, Cdk9, Smad3, p300 and DNaseI across ChIP-seq sumits of Cdk8, Cdk9 and Smad3. (c) ChIP-seq density plots centred around super-enhancers and typical enhancers defined by Med1. Flanking regions are 3 kb. (d) Box plot

shows the ChIP-seq density (rpm/bp) for Med1, Cdk8, Cdk9 and Smad3 in super-enhancers and typical enhancers defined by Med1. Box plot whiskers extend to 1.5x the interquartile range. (e) ChIP-seq binding profiles of Med1, Crdk8, Cdk9, Smad3, Oct4, Sox2 and Nanog at super-enhancer (mSE_00038) at the locus of Sox2 gene. (f) The heatmap of Med1, Crdk8, Cdk9 and Smad3 intensity at 231 mESC super-enhancers. (g) The left three scatter plot shows the Pearson's correlation of Med1 with Cdk8, Cdk9 and Smad3 respectively at the OSN regions. The right most scatter plot shows the Pearson's correlation between p300 and Smad3 at enhancer regions.

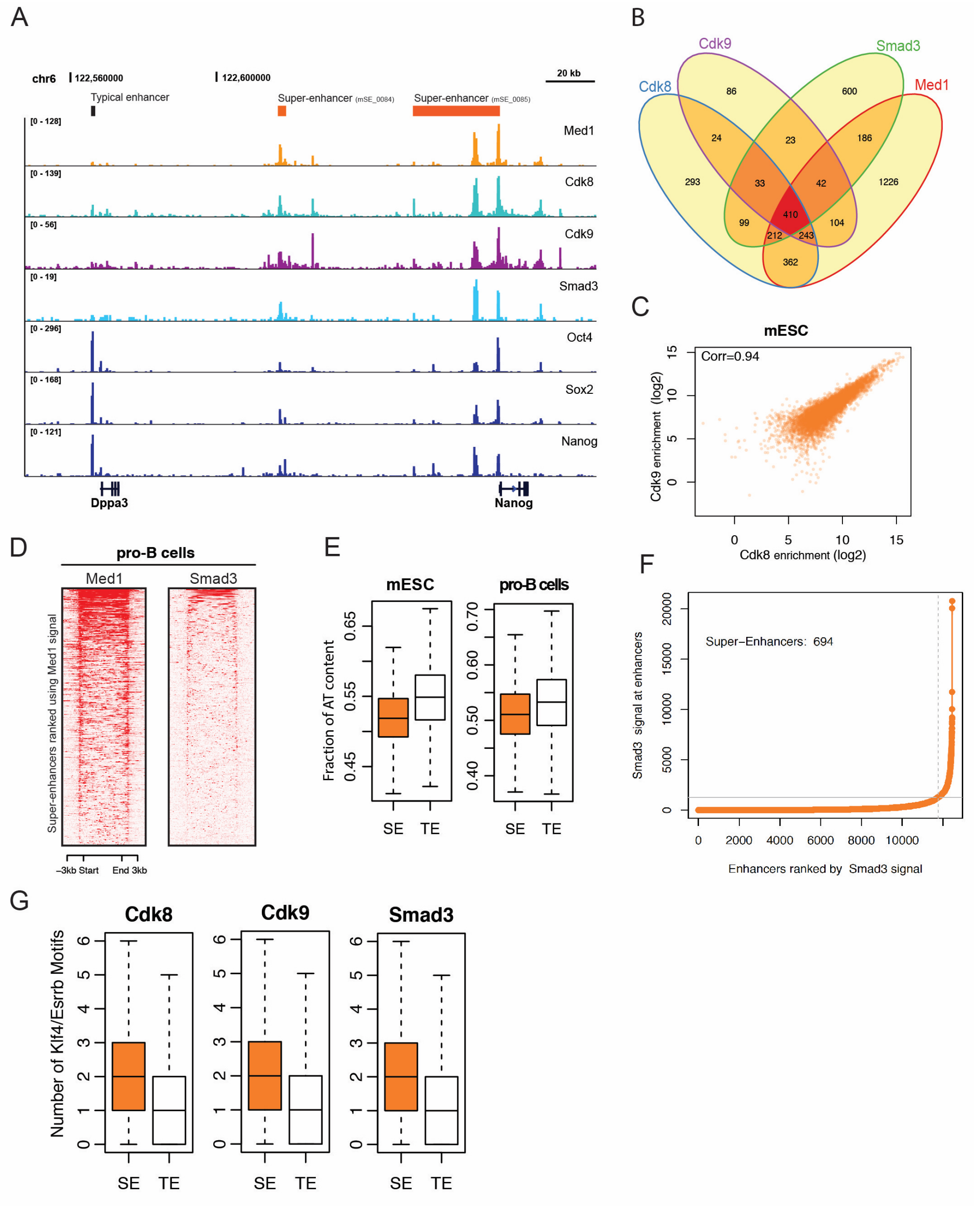

Figure S11: Characterization of super-enhancers and typical enhancers in mESC and pro-B cells. (A) ChIP-seq binding profiles of Med1, Crdk8, Cdk9, Smad3, Oct4, Sox2 and Nanog at the locus of Dppa3 and Nanog gene in mESC. (B) The overlap of ChIP-seq peaks for Cdk8, Cd9, Smad3 and Med1. (C) The Pearson's correlation of Cd8 and Cdk9 and mESC enhancers. (D) ChIP-seq binding profile of Med1 and Smad3 at super-enhancers ranked by Med1 in pro-B cells. (E) The fraction of AT-content at the constituents of super-enhancers and typical enhancers in mESC (p-value < 2.2e-16, Wilcoxon rank sum test) and pro-B cells (p-value < 2.2e-16, Wilcoxon rank sum test). (F) The hockey-stick plot shows the distribution of Smad3 ChIP-seq signal across the enhancers and separates the super-enhancers from typical enhancers where slope is 1. (G)Boxplot shows the number of motifs (Klf4/Essrb) found at the constituents of super-enhancers and typical enhancers defined by Cdk8, Cdk9 and Smad3 in mESC.

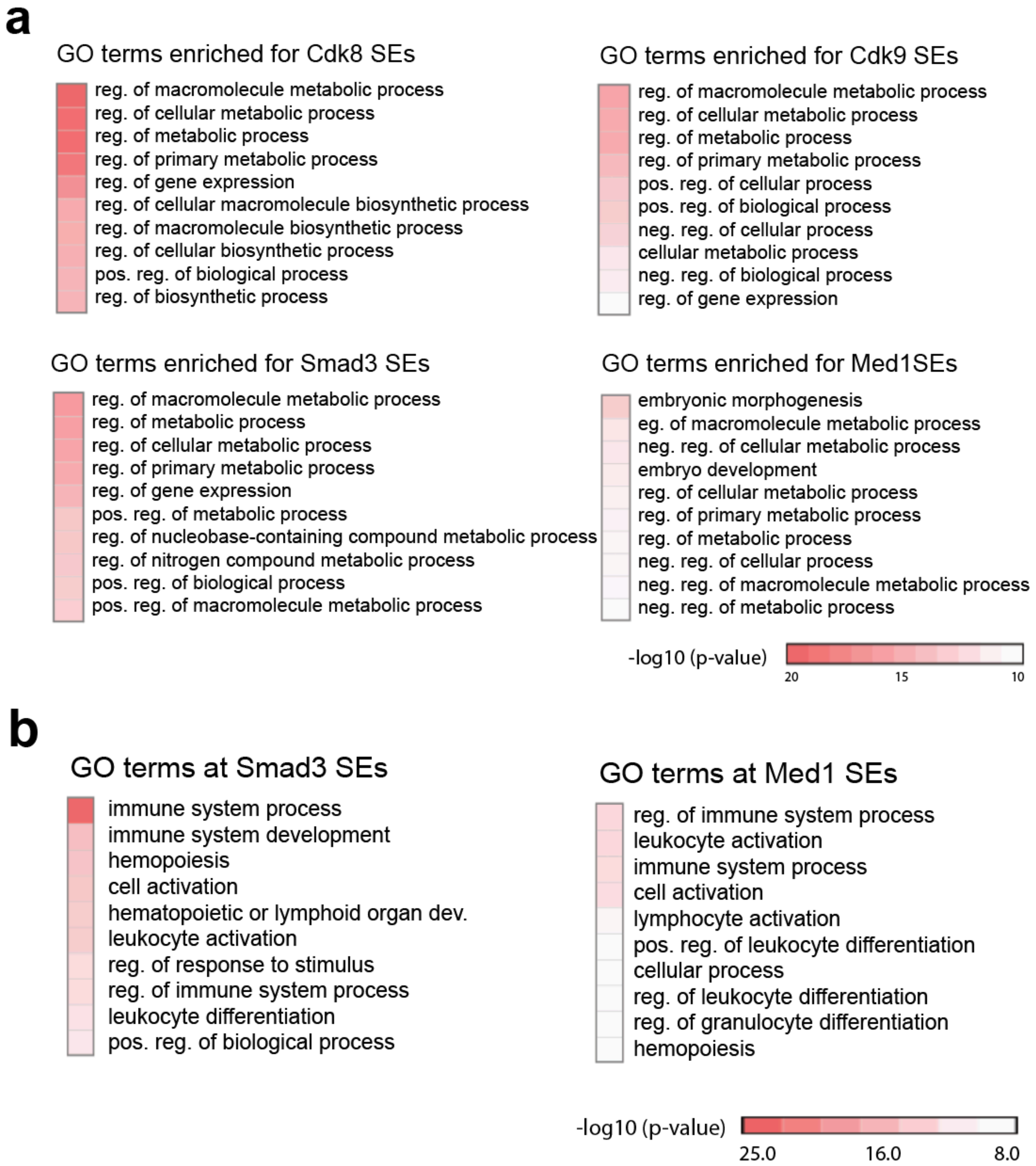

Figure S12: Gene Ontology (GO) enrichment analysis. (b) Gene ontology terms (Biological Process) for super-enhancers ranked by Cdk8, Cdk9, Smad3 and Med1 in mESC. (b) Gene ontology terms (Biological Process) for super-enhancers ranked by Med1 and Smad3 in pro-B cells.

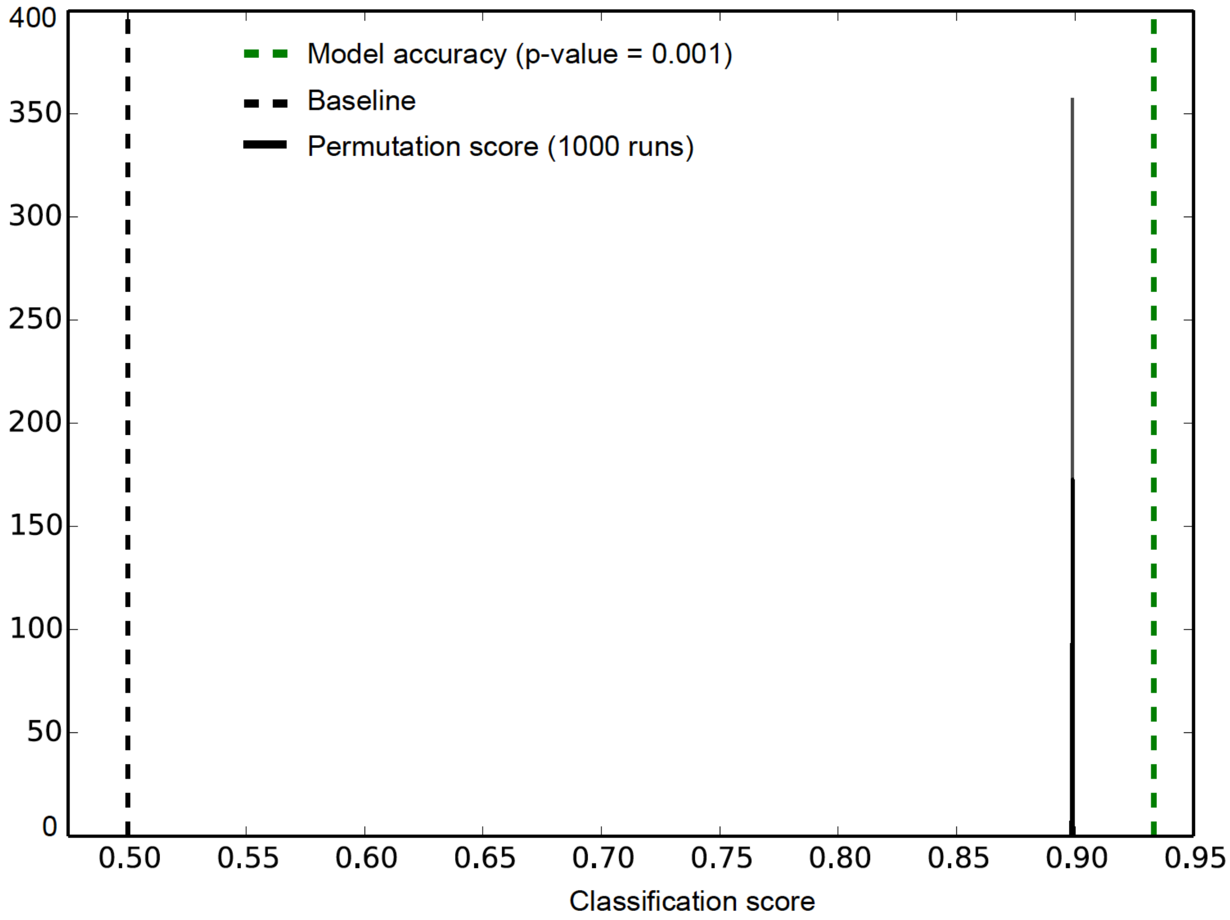

Figure S13: Plot shows the significance of increase in model AUC by using permutation test (1000 runs), which is statistically significant (p-value = 0.001). The p-value is calculated using Wilcoxon rank sum statistic.

Supplementary tables

Table S1: Comparison of the six models on each feature. The table shows precision, recall, F1-score and ROC. The table is ranked based on AUC score of Random Forest.

	Random Forest					Linear SVM					AdaBoost					k-NN					Naïve Bayes					Decision Tree				
Features	Precision	Recall	F1-score	AUC	PRC	Precision	Recall	F1-score	AUC	PRC	Precision	Recall	F1-score	AUC	PRC	Precision	Recall	F1-score	AUC	PRC	Precision	Recall	F1-score	AUC	PRC	Precision	Recall	F1-score	AUC	PRC
Brd4	0.76	0.77	0.77	0.85	0.86	0.81	0.7	0.75	0.91	0.91	0.83	0.59	0.69	0.91	0.9	0.78	0.68	0.72	0.86	0.87	0.79	0.72	0.75	0.9	0.9	0.79	0.72	0.75	0.91	0.9
H3K27ac	0.76	0.75	0.75	0.84	0.83	0.81	0.64	0.72	0.89	0.86	0.8	0.62	0.7	0.89	0.86	0.77	0.67	0.72	0.85	0.84	0.81	0.65	0.72	0.89	0.86	0.78	0.76	0.77	0.89	0.88
Cdk8	0.76	0.75	0.75	0.84	0.85	0.8	0.7	0.75	0.9	0.9	0.83	0.57	0.68	0.9	0.9	0.77	0.67	0.71	0.85	0.87	0.78	0.72	0.75	0.89	0.9	0.81	0.7	0.75	0.89	0.9
Cdk9	0.75	0.74	0.75	0.83	0.84	0.79	0.68	0.73	0.89	0.89	0.84	0.53	0.65	0.89	0.89	0.76	0.66	0.71	0.84	0.85	0.78	0.69	0.73	0.87	0.88	0.8	0.65	0.72	0.88	0.88
Med12	0.75	0.75	0.75	0.83	0.84	0.8	0.7	0.75	0.89	0.9	0.84	0.56	0.67	0.89	0.9	0.77	0.66	0.71	0.85	0.86	0.77	0.72	0.75	0.88	0.89	0.78	0.71	0.74	0.89	0.9
Pol2	0.74	0.74	0.74	0.82	0.82	0.81	0.62	0.7	0.87	0.85	0.81	0.61	0.69	0.88	0.86	0.76	0.65	0.7	0.84	0.83	0.75	0.62	0.68	0.8	0.82	0.79	0.67	0.72	0.87	0.86
p300	0.69	0.7	0.7	0.76	0.77	0.76	0.66	0.71	0.85	0.83	0.76	0.6	0.67	0.84	0.83	0.73	0.62	0.67	0.78	0.79	0.75	0.67	0.71	0.83	0.83	0.76	0.67	0.71	0.84	0.84
Lsd1	0.69	0.68	0.68	0.75	0.76	0.75	0.66	0.7	0.83	0.82	0.71	0.68	0.7	0.83	0.81	0.72	0.62	0.66	0.77	0.77	0.74	0.66	0.7	0.83	0.81	0.75	0.65	0.7	0.83	0.82
CBP	0.68	0.68	0.68	0.74	0.76	0.76	0.64	0.7	0.83	0.82	0.78	0.55	0.65	0.83	0.82	0.72	0.61	0.66	0.76	0.77	0.74	0.65	0.69	0.8	0.81	0.77	0.63	0.69	0.82	0.83
Nipbl	0.69	0.67	0.68	0.74	0.75	0.75	0.64	0.69	0.82	0.81	0.77	0.55	0.65	0.82	0.81	0.72	0.61	0.66	0.76	0.77	0.73	0.64	0.68	0.79	0.8	0.76	0.62	0.68	0.81	0.82
Smad3	0.66	0.67	0.67	0.72	0.74	0.74	0.64	0.68	0.8	0.8	0.79	0.49	0.61	0.8	0.8	0.71	0.6	0.65	0.74	0.75	0.71	0.64	0.68	0.77	0.79	0.76	0.61	0.68	0.8	0.81
H3K4me3	0.66	0.63	0.65	0.71	0.71	0.71	0.54	0.61	0.78	0.71	0.74	0.53	0.62	0.78	0.74	0.68	0.59	0.63	0.71	0.71	0.71	0.54	0.61	0.78	0.71	0.72	0.66	0.68	0.78	0.79
Smc1	0.65	0.64	0.64	0.7	0.71	0.71	0.61	0.66	0.78	0.74	0.73	0.58	0.65	0.78	0.75	0.69	0.59	0.63	0.71	0.72	0.71	0.62	0.66	0.78	0.74	0.7	0.69	0.7	0.78	0.78
Esrrb	0.64	0.63	0.63	0.69	0.69	0.72	0.58	0.64	0.78	0.74	0.72	0.55	0.63	0.78	0.74	0.69	0.58	0.63	0.72	0.7	0.71	0.58	0.64	0.76	0.73	0.7	0.65	0.67	0.77	0.76
Brg1	0.64	0.62	0.63	0.69	0.69	0.71	0.58	0.64	0.77	0.73	0.72	0.51	0.6	0.77	0.74	0.69	0.58	0.63	0.71	0.7	0.65	0.54	0.59	0.69	0.66	0.69	0.69	0.69	0.77	0.78
CHD7	0.63	0.62	0.63	0.68	0.68	0.68	0.62	0.65	0.76	0.71	0.67	0.65	0.66	0.76	0.73	0.68	0.58	0.63	0.7	0.7	0.67	0.62	0.64	0.75	0.71	0.68	0.69	0.69	0.76	0.76
Mi2b	0.63	0.61	0.62	0.67	0.68	0.68	0.62	0.64	0.76	0.71	0.69	0.59	0.64	0.76	0.72	0.67	0.57	0.62	0.68	0.68	0.63	0.58	0.61	0.67	0.65	0.69	0.66	0.68	0.75	0.76
H3K9me3	0.7	0.55	0.62	0.66	0.72	0.58	0.26	0.36	0.66	0.6	0.77	0.42	0.54	0.71	0.74	0.64	0.65	0.64	0.56	0.68	0.69	0.41	0.51	0.67	0.68	0.77	0.48	0.59	0.71	0.75
Tcfcp2l1	0.62	0.59	0.61	0.66	0.66	0.68	0.57	0.62	0.73	0.69	0.71	0.5	0.59	0.74	0.69	0.66	0.57	0.61	0.66	0.66	0.57	0.53	0.55	0.53	0.59	0.71	0.59	0.64	0.73	0.73
DNaseI	0.62	0.61	0.61	0.66	0.66	0.68	0.62	0.65	0.75	0.72	0.69	0.58	0.63	0.75	0.73	0.68	0.57	0.62	0.68	0.68	0.64	0.58	0.61	0.64	0.68	0.69	0.61	0.65	0.74	0.73
Klf4	0.62	0.6	0.61	0.65	0.65	0.67	0.56	0.61	0.74	0.68	0.67	0.53	0.59	0.74	0.67	0.67	0.56	0.61	0.68	0.66	0.67	0.56	0.61	0.74	0.68	0.68	0.63	0.66	0.74	0.73
Stat3	0.63	0.56	0.59	0.64	0.66	0.68	0.5	0.58	0.69	0.69	0.76	0.35	0.48	0.71	0.69	0.64	0.56	0.6	0.59	0.63	0.63	0.55	0.58	0.59	0.65	0.73	0.46	0.57	0.7	0.71
Nr5a2	0.62	0.59	0.6	0.64	0.65	0.67	0.54	0.6	0.72	0.68	0.68	0.47	0.55	0.72	0.68	0.65	0.56	0.6	0.65	0.65	0.67	0.54	0.6	0.72	0.68	0.68	0.64	0.66	0.72	0.73
HDAC2	0.6	0.59	0.6	0.64	0.65	0.66	0.6	0.63	0.73	0.69	0.68	0.58	0.63	0.73	0.69	0.66	0.56	0.6	0.64	0.65	0.61	0.57	0.59	0.63	0.64	0.68	0.62	0.65	0.73	0.72
Repeat fraction	0.61	0.6	0.6	0.64	0.64	0.55	0.42	0.48	0.48	0.59	0.63	0.73	0.68	0.71	0.66	0.65	0.56	0.6	0.64	0.64	0.63	0.53	0.57	0.68	0.64	0.65	0.78	0.71	0.68	0.78
GC content	0.6	0.58	0.59	0.63	0.63	0.6	0.58	0.59	0.68	0.61	0.6	0.78	0.68	0.69	0.64	0.65	0.55	0.59	0.64	0.63	0.63	0.59	0.61	0.69	0.64	0.62	0.77	0.69	0.69	0.74
HDAC	0.59	0.57	0.58	0.62	0.63	0.64	0.57	0.6	0.7	0.65	0.67	0.53	0.59	0.7	0.67	0.65	0.55	0.59	0.63	0.63	0.59	0.56	0.57	0.61	0.61	0.66	0.63	0.65	0.7	0.71
Prdm14	0.58	0.58	0.58	0.6	0.61	0.6	0.59	0.6	0.67	0.61	0.62	0.63	0.62	0.68	0.63	0.64	0.54	0.59	0.6	0.61	0.6	0.57	0.58	0.66	0.61	0.64	0.64	0.64	0.68	0.64
phastCons	0.56	0.55	0.56	0.58	0.59	0.42	0.47	0.45	0.47	0.45	0.58	0.75	0.65	0.66	0.61	0.62	0.53	0.57	0.59	0.59	0.59	0.56	0.58	0.64	0.6	0.6	0.69	0.64	0.64	0.6
H3K4me1	0.56	0.54	0.55	0.57	0.58	0.61	0.56	0.58	0.65	0.62	0.62	0.52	0.57	0.65	0.62	0.62	0.53	0.57	0.57	0.58	0.6	0.56	0.58	0.65	0.61	0.63	0.62	0.63	0.65	0.66
Oct4	0.54	0.54	0.54	0.55	0.57	0.54	0.55	0.55	0.58	0.55	0.58	0.53	0.56	0.6	0.58	0.61	0.52	0.56	0.55	0.56	0.51	0.49	0.5	0.49	0.51	0.6	0.54	0.57	0.58	0.57
Sox2	0.55	0.53	0.54	0.55	0.57	0.55	0.56	0.55	0.59	0.56	0.6	0.53	0.56	0.59	0.58	0.61	0.52	0.56	0.55	0.56	0.56	0.54	0.55	0.59	0.56	0.61	0.65	0.63	0.59	0.61
Tcf3	0.54	0.53	0.53	0.54	0.55	0.54	0.55	0.55	0.58	0.55	0.57	0.55	0.56	0.58	0.56	0.6	0.52	0.55	0.54	0.55	0.55	0.52	0.53	0.58	0.55	0.61	0.71	0.66	0.58	0.67
Nanog	0.53	0.51	0.52	0.53	0.56	0.64	0.49	0.55	0.49	0.65	0.6	0.57	0.58	0.58	0.58	0.61	0.51	0.55	0.53	0.55	0.48	0.48	0.48	0.47	0.48	0.63	0.59	0.61	0.57	0.61

Table S2: Comparison of the six models on each group/type of feature. The table shows precision, recall, F1-score and ROC. The table is ranked based on AUC score of Random Forest.

Features	Random Forest					Linear SVM					AdaBoost					k-NN					Naïve Bayes					Decision Tree				
	Precision	Recall	F1-score	AUC	PRC	Precision	Recall	F1-score	AUC	PRC	Precision	Recall	F1-score	AUC	PRC	Precision	Recall	F1-score	AUC	PRC	Precision	Recall	F1-score	AUC	PRC	Precision	Recall	F1-score	AUC	PRC
TFs	0.83	0.75	0.79	0.93	0.93	0.75	0.66	0.7	0.83	0.82	0.79	0.66	0.72	0.88	0.86	0.76	0.74	0.75	0.89	0.9	0.67	0.65	0.66	0.74	0.72	0.68	0.8	0.74	0.81	0.84
HMs	0.82	0.78	0.8	0.92	0.91	0.78	0.69	0.73	0.9	0.86	0.83	0.68	0.75	0.92	0.91	0.79	0.71	0.74	0.89	0.88	0.76	0.69	0.72	0.88	0.84	0.78	0.75	0.77	0.9	0.89
CRs	0.82	0.77	0.79	0.92	0.91	0.79	0.73	0.76	0.91	0.91	0.81	0.69	0.75	0.92	0.91	0.79	0.71	0.75	0.9	0.9	0.76	0.73	0.74	0.87	0.88	0.76	0.78	0.77	0.9	0.9
Mediator	0.8	0.75	0.77	0.89	0.89	0.8	0.71	0.75	0.91	0.91	0.84	0.6	0.7	0.9	0.91	0.78	0.68	0.73	0.87	0.88	0.77	0.78	0.77	0.9	0.91	0.82	0.7	0.75	0.9	0.9
Coactivators	0.74	0.69	0.72	0.83	0.82	0.77	0.67	0.71	0.85	0.85	0.78	0.62	0.69	0.86	0.85	0.74	0.64	0.69	0.81	0.82	0.75	0.7	0.72	0.84	0.84	0.76	0.69	0.72	0.85	0.85
P-TFEb	0.75	0.75	0.75	0.83	0.84	0.79	0.68	0.73	0.89	0.89	0.84	0.53	0.65	0.89	0.89	0.76	0.66	0.71	0.84	0.85	0.77	0.72	0.74	0.87	0.88	0.8	0.65	0.72	0.88	0.88
Lsd1-NuRD	0.72	0.69	0.71	0.81	0.8	0.75	0.66	0.7	0.83	0.82	0.76	0.63	0.69	0.84	0.82	0.73	0.63	0.68	0.79	0.8	0.73	0.65	0.69	0.81	0.79	0.75	0.67	0.7	0.83	0.83
Pol II	0.74	0.73	0.74	0.81	0.82	0.81	0.62	0.7	0.87	0.85	0.81	0.61	0.69	0.88	0.86	0.76	0.65	0.7	0.84	0.83	0.75	0.64	0.69	0.8	0.82	0.79	0.67	0.72	0.87	0.86
DNA Sequence	0.72	0.66	0.69	0.81	0.77	0.61	0.58	0.59	0.69	0.62	0.67	0.79	0.73	0.81	0.75	0.71	0.62	0.66	0.77	0.76	0.69	0.63	0.66	0.76	0.74	0.65	0.83	0.73	0.76	0.81
Cohesion	0.71	0.68	0.7	0.79	0.79	0.74	0.65	0.69	0.82	0.81	0.76	0.63	0.69	0.83	0.82	0.72	0.62	0.67	0.78	0.79	0.73	0.66	0.69	0.81	0.8	0.76	0.63	0.69	0.81	0.82
Motif	0.64	0.54	0.59	0.72	0.65	0.57	0.59	0.58	0.68	0.61	0.6	0.61	0.6	0.72	0.63	0.6	0.49	0.54	0.61	0.56	0.53	0.58	0.56	0.63	0.56	0.64	0.55	0.59	0.7	0.65
DNaseI	0.62	0.62	0.62	0.66	0.67	0.68	0.62	0.65	0.75	0.72	0.69	0.58	0.63	0.75	0.73	0.68	0.57	0.62	0.68	0.68	0.64	0.58	0.61	0.64	0.68	0.69	0.61	0.65	0.74	0.73

Table S3. Training the model on four cell-type data and test on one. The symbol ⊕ represents training set and ∅ represents test set. The table is ordered based on AUC score.

P493-6	*H2171*	*MM.1S*	*mESC*	*U87*	**Precision**	**Recall**	**F1-score**	**AUC**
∅	⊕	⊕	⊕	⊕	0.88	0.87	0.87	**0.95**
⊕	∅	⊕	⊕	⊕	0.85	0.84	0.84	0.92
⊕	⊕	∅	⊕	⊕	0.85	0.84	0.84	0.92
⊕	⊕	⊕	∅	⊕	0.82	0.82	0.82	0.90
⊕	⊕	⊕	⊕	∅	0.80	0.78	0.78	0.88

Public datasets used in this study

Table S4: ChIP-seq and DNase-seq data used to extract features, train model and perform feature analysis.

Factor type	Factor name	Purpose	Type	GEO ID
Transcription factors	Oct4, Sox2, Nanog, Esrrb, Klf4, Smad3, Tcfcp2l1, Prdm14, Stat3, Tcf3, Nr5a2	OSN co-bound enriched regions as training data	ChIP-seq	GSE44286,GSM288355 GSM288354, GSM623989, GSM53954
Histone modifications	H3K27ac, H3K4me1, H3K4me3, H3K9me3	Features	ChIP-seq	GSM594579, GSM281695 GSM307149, GSM18371
RNA polymerase II	RNA PolII	Features	ChIP-seq	GSM318444
Hypersensitive site	DNaseI	Features	DNase-seq	GSM1014154
Co-activators	p300, CBP	Features	ChIP-seq	GSM918750, GSM1246866
Chromatin regulators	Brg1, Brd4, Chd7	Features	ChIP-seq	GSM896923, GSM937540, GSM558674
Cohesion	Nipbl, Smc1	Features	ChIP-seq	GSM560350, GSM560342
Mediator complex	MED1	Define SEs	ChIP-seq	GSM560348, GSM560345
Mediator complex	MED12	Features	ChIP-seq	GSM560348, GSM560345
Lsd1-NuRD complex	Lsd1, Mi2b	Features	ChIP-seq	GSM687282, GSM687284

Table S5: Independent ChIP-seq data for MED1, H3K27ac and BRD4 in four human cell-types used for model validation

Cell-type	Source	MED1	BRD4	H3K27ac	Input
P493-6	B-cell lymphoma	GSM894078	GSM1036401	GSM894062	GSM894093
MM1.S	Multiple myeloma	GSM894109	GSM1033753	GSM894083	GSM894087
H2171	Small Cell Lung Carcinoma	GSM894081	GSM1038270	GSM894067	GSM894105
U87	Glioblastoma	GSM894082	GSM1038284	GSM894065	GSM894096

Table S6. ChIP-seq data used for prediction and feature ranking in pro-B cells.

Factor name	GEO ID
**Med1**	**GSM1038263**
**PU.1**	**GSM539538**
**Foxo1**	**GSM546525**
**Smad3**	**GSM539545**
**Ebf1**	**GSM1296532**
**p300**	**GSM987808**
**H3K27ac**	**GSM594592**
**H3K4me1**	**GSM594591**
**H3K4me3**	**GSM594594**
**DNaseI**	**GSM932972**
**Pol2**	**GSM1156660**
**Input/IgG**	**GSM539550**
